# HIV capsids: orchestrators of innate immune evasion, pathogenesis and pandemicity

**DOI:** 10.1099/jgv.0.002057

**Published:** 2025-01-13

**Authors:** Kate L. Morling, Mohamed ElGhazaly, Richard S. B. Milne, Greg J. Towers

**Affiliations:** 1 Division of Infection and Immunity, UCL, London, WC1E 6BT, UK

**Keywords:** capsid, cofactors, HIV, innate immunity, Vpr, Vpx

## Abstract

Human immunodeficiency virus (HIV) is an exemplar virus, still the most studied and best understood and a model for mechanisms of viral replication, immune evasion and pathogenesis. In this review, we consider the earliest stages of HIV infection from transport of the virion contents through the cytoplasm to integration of the viral genome into host chromatin. We present a holistic model for the virus–host interaction during this pivotal stage of infection. Central to this process is the HIV capsid. The last 10 years have seen a transformation in the way we understand HIV capsid structure and function. We review key discoveries and present our latest thoughts on the capsid as a dynamic regulator of innate immune evasion and chromatin targeting. We also consider the accessory proteins Vpr and Vpx because they are incorporated into particles where they collaborate with capsids to manipulate defensive cellular responses to infection. We argue that effective regulation of capsid uncoating and evasion of innate immunity define pandemic potential and viral pathogenesis, and we review how comparison of different HIV lineages can reveal what makes pandemic lentiviruses special.

## Introduction: the HIV capsid – the metastability issue

**Fig. 1.**
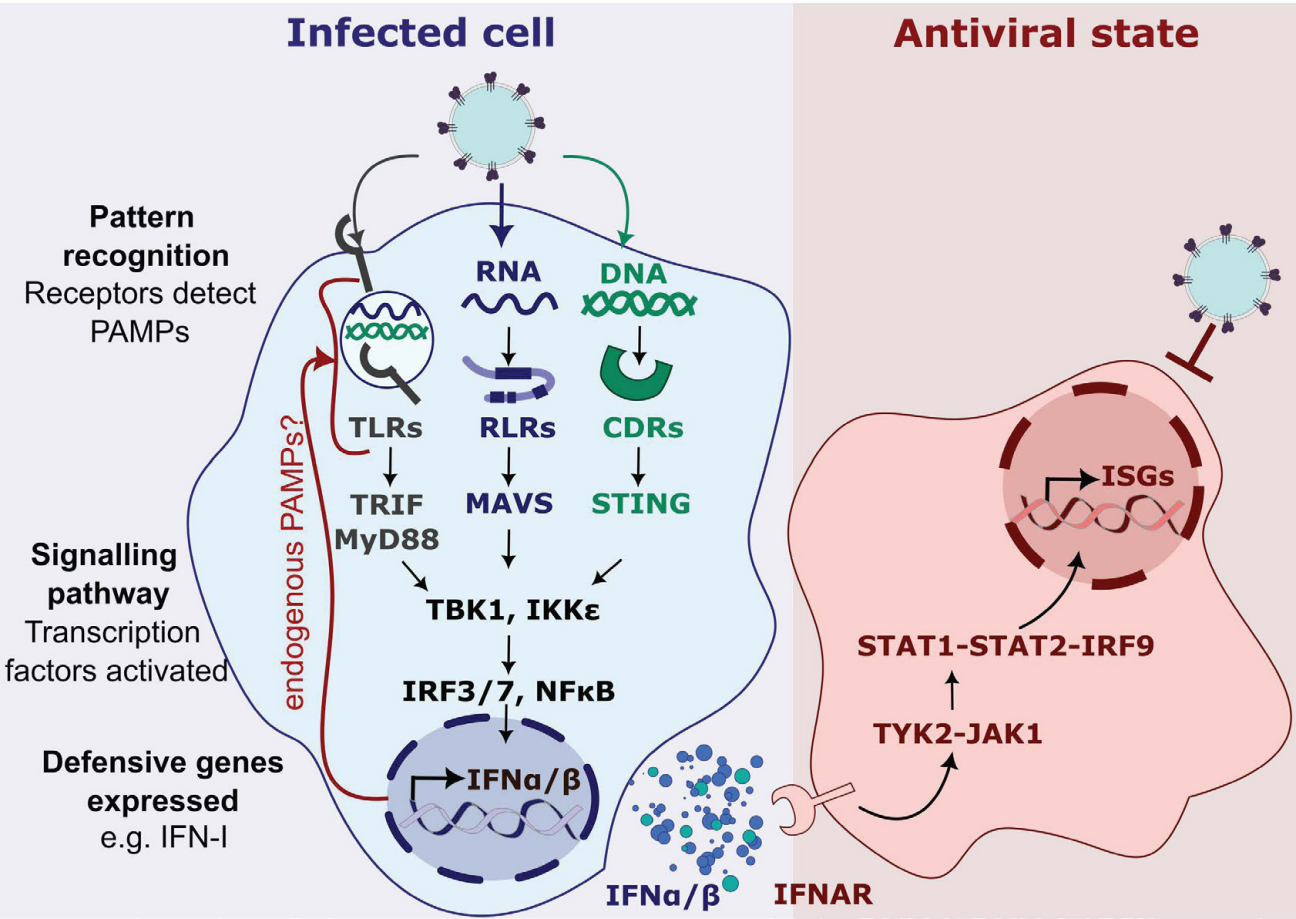
A simple model of innate immune sensing. In a simple model, pathogen-associated molecular patterns (PAMPs) including nucleic acids, lipoproteins and glycoconjugates, for example, lipopolysaccharides (LPS), are recognized by pattern recognition receptors (PRRs) including Toll-like receptors (TLRs), RIG-I-like receptors (RLRs) and cytoplasmic DNA receptors (CDRs) [210,211]. TLRs are membrane bound and typically recognize PAMPs on the cell surface or within endosomes. RLRs such as RIG-I and MDA5 sense RNA. CDRs such as the cyclic GMP–AMP synthase (cGAS) detect DNA. Upon activation, PRRs converge on activating downstream kinases including TBK1 and IKKε, resulting in the phosphorylation of a variety of transcription factors, particularly IFN regulatory factors (IRFs) and nuclear factor kappa-light-chain-enhancer of activated B cells (NFκB). These translocate to the nucleus and initiate transcription of around 500 defensive genes including type I IFN (IFN-I) and inflammatory cytokines, which regulate adaptive immune responses [212,213]. IFNs are secreted from the infected cell to act in a paracrine or autocrine manner by binding to IFN receptors and activating the JAK/STAT pathway, which leads to the expression of IFN-stimulated genes (ISGs). ISG expression establishes an antiviral state in the tissue microenvironment, in which the infection-resistant cells can suppress the spread of the virus [214]. Of course, viruses have evolved to subvert these defensive processes including evasion mechanisms (hiding PAMPs) and direct antagonism of IFN signalling and ISG activities. But we argue that nonetheless, all viruses will trigger IFN to some degree and all viruses are sensitive to IFN to some degree. Importantly, if these inflammatory responses do not suppress infection, they drive disease. Thus, we argue that the frequency of successful transmission and all aspects of viral pathogenesis relate back to this interaction with defensive processes in the infected cells. Importantly, recent evidence suggests sensing of disturbed cell biology during infection, for example, expression of endogenous PAMPs during influenza infection, or after LPS exposure [215,216], or intronic sequences appearing in the cytoplasm in cancer [217]. Thus, the distinction between self and non-self becomes blurred as mechanisms of sensing are uncovered and PAMPs associated with nucleic acid sensing may be derived from self.

Like other enveloped viruses, human immunodeficiency virus (HIV) enters cells by fusing target cell and viral membranes through interactions between the viral envelope protein (gp160) and cellular receptors (CD4 and CXCR4 or CCR5). *In vivo*, HIV productively infects T-cells with some HIV also infecting macrophages. Fusion delivers the viral capsid and associated proteins into the hostile environment of the cytoplasm. Hostility derives from the intracellular, also called the intrinsic, innate immune system, which is the ability of cells to detect and then react to infection, making an antiviral response that can stop a virus dead in its tracks. A simple model for innate immunity is presented in Fig. 1.

**Fig. 2.**
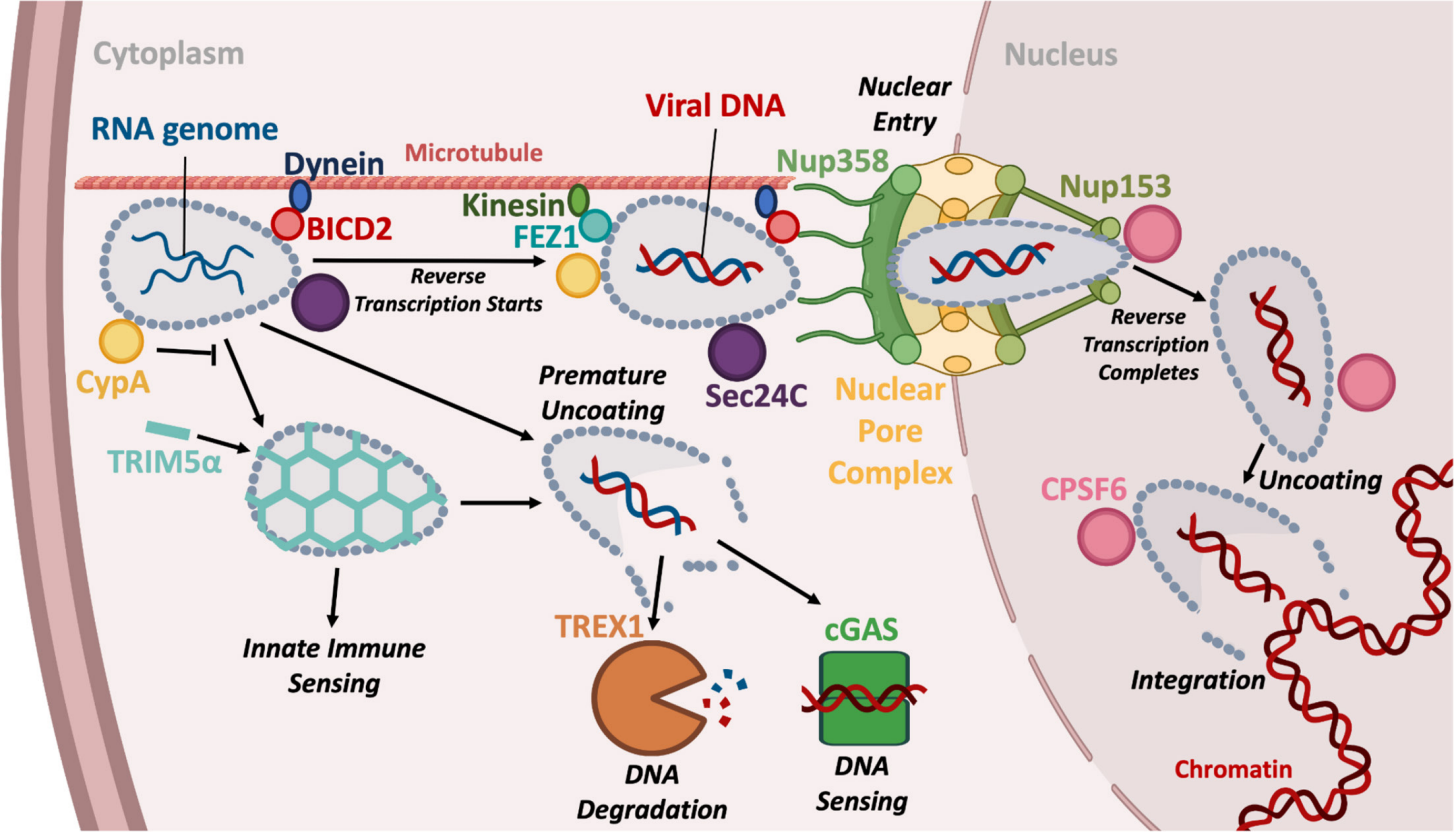
Early events in the HIV life cycle. Upon fusion with the plasma membrane, the viral capsid is released into the cell. It then travels across the cytoplasm to the nuclear pore and into the nucleus. RT, which generates viral DNA from the RNA genome, occurs within the capsid during this journey. The capsid maintains reverse transcriptase enzyme and RNA template in close proximity to drive RT. Once in the nucleus, likely in contact with chromatin, capsid uncoating occurs, releasing nascent viral DNA for integration. Encapsidated DNA synthesis protects viral nucleic acids from detection by cytosolic nucleic acid sensors including cGAS and degradation by nucleases including TREX1. Throughout its journey within the cell, capsid interacts with host cofactors including microtubule and NPC components, which regulate trafficking and capsid stability to ensure that uncoating occurs at the appropriate time and place. Disturbing cofactor interactions can lead to premature uncoating, DNA sensing and an innate immune response. Created using BioRender.

**Fig. 3.**
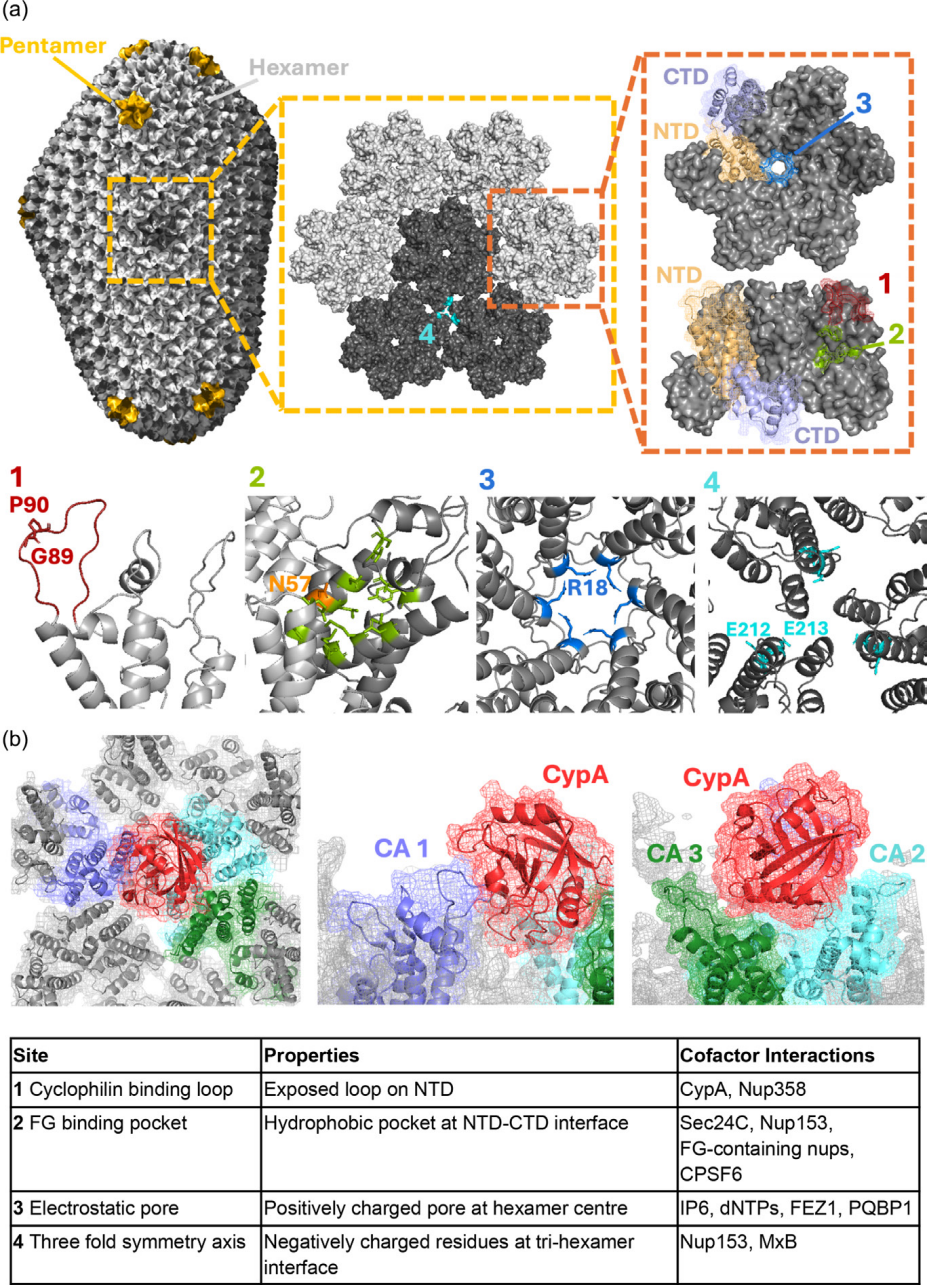
HIV capsid structure and cofactor binding sites. (**a**) The HIV-1 capsid is formed of around 250 hexamers (grey) and exactly 12 pentamers (yellow) of CA protein, which pack together to form a cone-shaped structure that encases the viral genome and safeguards RT in the cytoplasm and nucleus. Monomeric CA protein is formed of two, predominantly alpha-helical domains, an N-terminal domain (NTD) (orange) and a C-terminal domain (CTD) (purple), which are connected by a flexible hinge. The NTDs arrange in a ring at the centre of CA pentamers/hexamers, encircled by an outer ring of the CTDs [255]. Larger lattices assemble through dimeric and trimeric interactions between CTDs of adjacent hexamers and pentamers, with pentamers concentrated at each end of the cone to facilitate curvature. There are four well-established cofactor binding sites on capsid: the cyclophilin A (CypA)-binding loop (red), the FG-binding pocket (green), the electrostatic pore (blue) and the threefold symmetry axis (teal). Cone structure is rendered using cellPACK [256]. (**b**) A single CypA molecule (red) can simultaneously interact with multiple CA monomers, forming both canonical (purple) and non-canonical (cyan and green) interactions [30]. PDB (6Y9W) DOI: https://doi.org/10.2210/pdb6Y9W/pdb. Made using PyMOL.

For HIV, the general idea is that the conical capsid is a molecular machine, built of viral CA protein, that has evolved to regulate infection whilst avoiding the activation of innate immune sensors and the ensuing antiviral responses. Capsids contain, protect and regulate the synthesis of viral DNA by reverse transcription (RT) (Fig. 2). During this process, capsids travel across the cytoplasm, likely regulated by interaction with microtubules, through nuclear pore complexes (NPCs) to active chromatin where they disassemble, referred to as uncoating, and integrate viral DNA into chromatin. The challenge for viral capsids is to evolve a mechanism of highly regulated metastability. The capsid must be stable up until the point where it should uncoat and then it should become unstable at exactly the right time and in the right place. Early uncoating leads to viral genome degradation, integration into genomic regions less compatible with replication or, worse, activation of innate immune sensing, if a prematurely released genome is detected by DNA sensors such as the cyclic GMP–AMP synthase (cGAS). We hypothesize that the HIV capsid regulates metastability through interaction with host cofactors. There are currently four well-defined capsid sites that interact with host proteins (Fig. 3, Box 1). We propose that through these interactions, HIV reacts to its location, allowing uncoating at precisely the right time and in the right place, to maximize infection potential and avoid innate immune activation. Importantly, host cofactors are not the only proteins that recruit to incoming HIV capsids. Capsids are also targeted by defensive host proteins called restriction factors as reviewed in [1]. Once uncoating is triggered, the viral genome is rapidly integrated into the host chromatin to form the provirus, catalysed by the viral integrase. HIV favours integration into transcriptionally active regions near the nuclear periphery guided by host cofactors LEDGF/p75 and cleavage and polyadenylation specificity factor 6 (CPSF6) [2,6]. The proviral 5′ HIV LTR acts as a promoter driving viral gene transcription by host RNA polymerase II [7]. Notably, the recruitment of epigenetic regulators including histone deacetylases and methyltransferases leads to transcriptional silencing of a subset of proviruses, leading to a latent infected state. Latently infected T-cells are important because they are resistant to antivirals and immune clearance because they do not make viral proteins. But if antiviral treatment is stopped, they gradually reseed the infection as they get activated by natural processes to express the virus thereby preventing cure [8,9]. Discovery of antagonistic interactions between host epigenetic regulator complexes such as human silencing hub (HUSH) and lentiviral accessory proteins Vpx and Vpr suggests complex regulation of viral gene expression by virus and host relevant to latency mechanisms and therapeutic cures [10,12], discussed later in this review.

Box 1: **There are four characteriszed cofactor binding sites on HIV capsids**

There are two types of HIV, both derived from multiple zoonoses: HIV-1 comes from chimpanzees and gorillas and HIV-2 is derived from sooty mangabeys. The vast majority of studies focus on HIV-1. The HIV-1 capsid is conical and built of around 250 hexamers and exactly 12 pentamers of the CA protein. There are five pentamers at the wide end and seven at the sharp end. Pentamers provide the required curvature for fully sealed capsid structures (Fig. 3a). We propose that this rather complex fullerene geometry could allow maximal diversity of CA–CA interactions, providing complex curvatures and geometries, which allow the most precise regulation of capsid metastability by host cofactor binding. This fullerene shape may also be important for capsid import through NPCs by driving directionality.

Although each cofactor binding site can recruit different cofactors, because capsids are built from many hexamers, there is plenty of room for all the cofactors to be recruited to the same viral capsid. Cofactor stoichiometry is poorly understood but one possibility is that sequential recruitment of cofactors gradually destabilizes the capsid with either genome synthesis or a final cofactor interaction or both, driving uncoating at precisely the right time and place. Another possibility is a handover model with sequential cofactors replacing each other as the infection progresses. We expect that by studying the impact of cofactor binding at different capsid curvatures, we will be able to explain how cofactors achieve the necessary uncoating precision.

**The cyclophilin-binding loop**

The peptidyl-prolyl isomerase cyclophilin A (CypA) mediates complex effects on viral capsid behaviour. CypA recruitment regulates capsid stability [30,218], restriction factor sensitivity [28,219] and the impact of downstream cofactor binding, we hypothesize, by regulating dynamic allostery [114,122, 132]. CypA targets a flexible loop on the HIV-1 CA N-terminal domain (NTD) surface, with CA residues G89 and P90 burying in the CypA active site [220,224]. Hexamer and lattice curvature vary across the surface of the viral capsid, and CypA appears to sense specific curvatures and geometries by binding in different modes as curvature varies [30,123, 225]. Cryo electron microscopy (cryoEM) structures of CA tubes have shown that CypA molecules contact three CA monomers in the lattice, one at the canonical CypA-binding loop and two at non-canonical sites across the dimer and trimer interfaces between hexamers, providing a mechanism for viral capsid stabilization (Fig. 3b) [30]. In addition, atomic force microscopy (AFM) on CA assemblies showed that CypA increases capsid ‘stiffness’, meaning greater force is needed to deform the CA structure, indicating that CypA strengthens interactions between CA monomers in the lattice [225]. Consistent with this, CypA delays the uncoating of isolated capsid cores *in vitro* [90,226]. In humans, CypA protects the pandemic HIV-1 group M capsid from restriction by TRIM5α [28], as discussed later. In a simple model, CypA simply gets in the way of TRIM5α binding (Fig. 2). However, rhesus macaque TRIM5α targets HIV more effectively in the presence of CypA, suggesting a more complex model [32,227].

The HIV-1 CA CypA-binding loop is also bound by the C-terminal cyclophilin homology domain of nucleoporin 358 (Nup358) in the outer nuclear pore. This may be important for orienting the cone-shaped capsid to go into the NPC sharp end first, as is observed in cryoEM studies [61] (Fig. 2). Certainly, Nup358–CA interactions are important for efficient nuclear transport and integration site targeting [4,228, 229] and may be important for viral capsid trafficking in the cytoplasm [56]. Both CypA and Nup358 catalyse CA P90 *cis*/*trans* peptide isomerization, but the importance of this for HIV infection remains unclear because isomerization cannot be separated from CypA binding [4,228, 229].

**The FG-binding pocket**

The NPC forms a selective permeability barrier comprising phenylalanine-glycine (FG) motif repeats contributed by FG-bearing nucleoporins (nups). Viral capsids form low-affinity interactions with the FG nups via a hydrophobic FG-binding pocket at the CA NTD–C-terminal domain (CTD) interface between two monomers in the same hexamer, with N57 being essential for binding [82,83]. This facilitates the phase separation of viral capsids into the permeability barrier, mediating karyopherin-independent import into the nucleus [82,83]. The FG-binding site also recruits cytoplasmic and nuclear FG-bearing cofactors to regulate infection, with these cofactors acting as waypoints to guide capsid to chromatin. In the cytoplasm, the CA FG-binding site is bound by Sec24C, at the inner nuclear pore by nucleoporin 153 (Nup153), followed by nuclear S/R family protein, CPSF6 in the nucleus [14,80, 84, 93, 97, 98, 141, 230] (Fig. 2). In addition to unique interactions from each cofactor, the phenylalanines conserved in Sec24C, Nup153 and CPSF6 all orient the main chain to form hydrogen bonds with CA N57 [93,141]. Notably, N57 mutations are the only CA mutations that impact nuclear transport in cell lines, evidenced by reduced infection, particularly in cell-cycle arrested cells [82,84, 85]. In Sec24C, Nup153 and CPSF6, the FG region is flanked by prion-like, disordered low complexity regions (LCRs), which are important for high affinity binding to CA tubes [231]. There is evidence that LCR–LCR interactions facilitate CPSF6 assembly in a zigzag arrangement on capsid lattice, templated by interactions with the FG site [231]. Recent cryoEM structures of peptides derived from Sec24C, Nup153 and CPSF6 in complex with HIV-1 capsids show that, like CypA, FG cofactors bind specific locations on the curved lattice surface, with Nup153 preferring more curved regions [232]. The FG cofactors do not seem to be able to bind CA pentamers due to steric clashes with the somewhat different pentamer pocket [110,232]. The depletion of Sec24C reduced capsid stability in cells, and a GST-Sec24C_196-314_ fragment bearing the FG-motif stabilized isolated capsids and assembled CA tubes in biochemical assays, suggesting that, like CypA, Sec24C also contributes to capsid core stability [141]. In addition, Sec24C depletion reduced infectivity, particularly in non-dividing cells, suggesting a role in viral capsid transport into the nucleus [141]. The nuclear FG cofactor CPSF6 has an intriguing but poorly understood role. It appears to pull capsids through nuclear pores by competing with FG nups for the FG-binding site from the nuclear side of the NPC [91]. Strikingly, CPSF6 is disordered and in the presence of capsid forms phase-separated puncta, which colocalize with nuclear speckles [62,63, 99, 100, 107, 233, 234]. CPSF6 recruitment seems to take capsids into the phase-separated speckles and target integration into speckle-associated chromatin domains (SPADs) [88,99, 100, 107, 140]. Disturbing CPSF6 interactions retargets HIV integration [4,5] and tends to cause the virus to activate innate immune sensing [14]. Thus, recruitment into CPSF6 phase-separated speckles both targets integration and contributes to innate immune evasion. CPSF6’s ability to form biomolecular condensates has been attributed to its intrinsically disordered, C-terminal arginine-rich mixed charge domains as well as its central prion-like LCRs [101,235, 236].

The FG-binding site is also the target of the Pfizer experimental capsid inhibitor PF74 and the Gilead first-in-class ultrapotent capsid targeting drug lenacapavir (LEN), which contain phenyl (PF74) and difluorobenzyl (LEN) moieties that superimpose with the FG motif in the crystal structures of the host cofactors [93,97, 98, 116, 237, 238]. Both of these inhibitors compete with FG cofactors for binding to HIV-1 capsids and cause premature capsid uncoating, leading to the inhibition of RT [90,91, 93, 97, 116, 117, 238]. They also both inhibit HIV-1 particle production [116,237].

**The electrostatic capsid pore**

HIV DNA synthesis takes place inside the capsid, with encapsidated RT fuelled by the import of nucleotides through a dynamic pore at the centre of each CA hexamer and pentamer [144] (Fig. 3a). This pore contains six positively charged arginine residues (CA R18), which recruit the negatively charged nucleotides. However, the R18 ring experiences destabilizing electrostatic repulsion due to the concentrated positive charge, and this is greater in pentamers than in hexamers [239]. Crucially, the negatively charged cellular metabolite IP6 is recruited to capsid pores, neutralizing R18 monomer–monomer repulsion to stabilize CA hexamers, pentamers and capsids in general [240,241]. Indeed, it has been suggested that IP6 binds more tightly to pentamers than hexamers [123]. The discovery that IP6 stabilizes pentamers explains how it facilitates *in vitro* capsid cone assembly because IP6 driving cone formation is consistent with it favouring pentamer formation and thus cone closure. Flexibility at the trimeric hexamer interfaces and conformational switches within CA monomers also contribute to pentamer incorporation and lattice curvature [232]. Specifically, a TVGG motif in CA’s NTD adopts pentamer- and hexamer-specific folds, and IP6 may allosterically trigger a conformational switch to promote pentamer formation [110,232]. IP6 is recruited into capsids via binding to CA K158 and K227 during assembly of the immature CA lattice. On Gag cleavage and cone formation, IP6 is recruited to R18 and K25 in the hexamers and pentamers [110,112, 123, 240,244]. Importantly, not all CA protein within the immature virion is used to form mature viral capsids, ensuring an excess of both CA protein and IP6 to drive mature cone assembly. The ability to form authentic conical HIV-1 capsids using IP6 has transformed biochemical and structural studies of HIV capsids [92,110, 123, 240, 244]. In previous work, hexamers were typically cross-linked by cysteine mutations A14C and E45C to stabilize them. However, cross-linked hexamers are flattened, and even uncross-linked hexamer tubes lack the physiologically relevant curvature found in cone-shaped HIV-1 capsids. In addition, E45C mutations interfere with critical hexamer–hexamer interactions [245], and viruses with CA A14C and E45C are non-infectious [234], making them a poor model for studying CA structure–function relationships.

The R18 capsid pore inevitably binds negatively charged peptides due to its highly positively charged nature. For example, kinesin-1 adaptor protein, FEZ1, has been suggested to bind the pore via negatively charged poly-glutamate motifs and promote capsid trafficking along microtubules [52,53] (Fig. 2). Polyglutamine-binding protein 1 (PQBP1) has also been described to bind the R18 ring via negatively charged residues at its N-terminus [246,247]. This has been described as allowing cGAS to sense the genome, but the details of how PQBP1 recruitment links to genome exposure and cGAS activation are not yet clear [247,248].

**The three-fold symmetry axis**

Most recently, a fourth capsid cofactor binding site has been defined. In addition to its interactions with the FG-binding pocket, biochemical studies suggest that Nup153’s C-terminal RRR motif can form electrostatic interactions with glutamic acid residues (E75, E212 and E213) at CA’s C-terminus in a pocket at the threefold symmetry axis at the interface between three CA hexamers [249]. Similar biochemical studies have suggested that restriction factor MxB also binds the tri-hexamer interface via the triple arginine (RRR) motif at its disordered N-terminus [250]. This MxB motif is essential for the inhibition of HIV-1 infection [251] and nuclear transport [219,252,254].

We argue that the capacity of an incoming virus to evade innate sensing determines the transmission frequency, pathogenesis and pandemic potential. That is, the contents and behaviour of the virus particles entering target cells at transmission dictate everything that follows. We therefore discuss the role of capsid and accessory proteins Vpr/Vpx, the two components of the virion that have explicitly evolved to evade and antagonize innate immunity to promote transmission.

## Capsids remain intact in the cytoplasm to protect viral DNA from innate immune detection

Early work suggested that HIV capsids break apart soon after cell entry to facilitate infection [13]. These conclusions are probably explained in part by the loss of capsid integrity due to the loss of IP6 during purification. After the discovery of the role of IP6 in stabilizing capsids, intact capsid purification with high IP6 concentrations is relatively tractable. We proposed that rather than uncoating in the cytoplasm, capsids remain intact to protect the process of viral DNA synthesis from the hostile cytoplasmic environment [4,14, 15]. If HIV capsids uncoat in the cytoplasm, released viral DNA is expected to activate DNA sensor cGAS. cGAS activation leads to the production of a second messenger cGAMP, which is detected by stimulator of IFN gene (STING), triggering IFN-I production and an antiviral response [16,19]. cGAS can be activated by HIV-1, the most common type of HIV, which has been mutated in the Gag cleavage sites to form defective viral capsids, or by inhibitors that break capsids apart. For example, disrupting capsid formation with protease inhibitors, *gag* cleavage mutations (L363I M367I), or capsid destabilizing inhibitors PF74 or lenacapavir (LEN), leads to premature release of viral DNA, cGAS activation and an IFN response [20,23]. Similarly, *gag* mutations, which prevent packaging of IP6 (K158A), result in defective capsids, again causing cGAS-dependent innate immune sensing [24].

## A role for three prime repair exonuclease 1

Despite being part of the innate immune system, the three prime repair exonuclease 1 (TREX1) plays a counterintuitive role as a cofactor, at least for the WT pandemic HIV-1(M) lineage virus [25]. Differences with non-pandemic HIVs are discussed later. TREX1 degrades HIV DNA that escapes from capsids that uncoat spontaneously or are uncoated by poorly understood defensive processes within the cell [25]. TREX1 depletion therefore causes innate immune activation in HIV-1(M)-infected cells via cGAS sensing of accumulated escaped viral DNA [23]. The 3′ processing of full-length reverse-transcribed viral DNA by integrase, which is essential for integration, renders the genome resistant to the 3′ exonuclease activity of TREX-1 [26]. Thus, it seems that the virus has evolved to use TREX1 to degrade DNA released from early uncoating capsids, whereas integrase-processed DNA from timely nuclear uncoating escapes TREX1 activity [27]. TREX1 therefore enhances infection by limiting innate immune activation by cGAS. We assume a threshold in which WT HIV-1(M) capsids do not release enough DNA to overwhelm TREX1 but where capsid is mutated, or PF74 inhibited, enough DNA is released to overcome TREX1 and activate cGAS. As with all sensing events, activation is dose-dependent and we propose that WT HIV-1(M) infection does not typically activate cGAS unless high infection doses are used [16].

## Evidence for cofactor regulation of capsid integrity

We argue that a key role of capsid-binding host cofactors is regulating capsid integrity to avoid cGAS activation and/or genome degradation. Capsid mutations, which prevent interactions with CPSF6 (N74D) or CypA/Nup358 (P90A), shRNA depletion of CPSF6 or pharmacological inhibition of CypA recruitment, all lead to IFN-*β* production and innate immune recognition of HIV-1, which prevents replication in primary monocyte-derived macrophages (MDMs) [14,20]. In this work, IFN induction requires RT, supporting the notion that viral DNA is sensed, although a role for cGAS is not well evidenced for these cofactor binding mutants [14] and there is certainly a role for TRIM5*α* in detecting CypA-binding mutants, as discussed in the following section [28,29].

## TRIM5α and CypA

How cofactors regulate capsid stability is incompletely understood. In the cytoplasm, CypA coats incoming capsids (Fig. 2), and *in vitro*, recombinant CypA delays the uncoating of isolated capsids likely through bridging hexamers, as discussed in Box 1 [30, 26–28]. Recent cryo electron tomography (cryoET) studies suggest that CypA is stripped off capsids as they enter nuclear pores, suggesting that CypA function is strictly cytoplasmic [31]. CypA protects capsids from TRIM5*α* [28,29], a central HIV restriction factor that inhibits retroviral replication in a species-specific way [32,36]. Target specificity is determined by the sequence of the TRIM5*α* C-terminal PRY/SPRY capsid-binding domain [37,40]. TRIM5*α* recognizes the curved capsid lattice, spontaneously oligomerizing into a hexagonal ‘cage’ over the capsid surface [41,43]. ‘Caging’ activates TRIM5*α* RING E3 ubiquitin ligase activity. When auto-monoubiquitinated at its N-terminus TRIM5*α* is turned over by the proteasome, however, once the hexagonal TRIM5*α* cage is formed, triggered by the presence of capsid, the TRIM5*α* RING domains become activated through dynamic dimerization at the trimeric interface of the TRIM5*α* CA-bound cage [44]. TRIM5*α* auto-polyubiquitinates with complex K63-linked ubiquitin chains [44], which enhances TRIM5*α*-mediated activation of antiviral innate immune signalling and simultaneously stimulates proteasome-dependent destruction of the associated capsid [44,49]. Given that CypA protects against TRIM5*α*, cell line/type differences in CypA dependence may reflect TRIM5*α* levels or innate signalling functionality. Interestingly, phenylalanine-glycine (FG)-binding site mutation N74D sensitizes HIV-1 to TRIM5*α*, consistent with allostery between the CypA and FG-binding sites [50]. This is consistent with our central hypothesis that serial cofactor interactions each impact the consequence of downstream cofactor interactions through dynamic allostery to provide directionality and precise regulation of uncoating.

## Capsid trafficking on microtubules

There is evidence that HIV capsids traffic along microtubules towards the nucleus, reviewed in [51]. This is likely facilitated by microtubule-associated motor proteins including kinesin-1 adaptor protein FEZ1 and dynein adaptor protein and activator BICD2 (Fig. 2). BICD2 binds CA via a C-terminal CC3 cargo-binding domain and FEZ1 binds the CA pore [52,55]. Notably, the depletion of BICD2 during HIV infection causes IFN-stimulated gene (ISG) induction, consistent with a role in preventing premature capsid uncoating or protection from TRIM5*α* [55]. Nup358, which is classically associated with the outer NPC, may also be associated with microtubules during HIV-1 infection and therefore may contribute to capsid transport to the nuclear pore [56]. Microtubule association could facilitate the efficient trafficking of intact capsids through the cytoplasm to nuclear pores, as well as contributing to capsid stability thereby preventing viral genome release and innate immune sensing.

## Intact capsids are transported through nuclear pores

The unique capacity of lentiviruses to traverse nuclear pores and thus infect both dividing and non-dividing cells has long been associated with capsid [57,58], but for many years, it was thought that intact HIV capsids, which are 60 nm wide, are too large to fit through the NPC (determined to have a 40 nm diameter in cryoEM structures of isolated nuclear envelopes) [59,60]. However, recent cryoEM studies show intact capsids traversing NPCs and within the nucleus. In these studies, capsid uncoating (but not full disassembly) and genome release can be seen close to integration sites [61,70]. In some studies, NPCs of infected and non-infected T-cells and macrophages appeared dilated compared to isolated nuclear envelopes, with a diameter sufficient to accommodate intact capsid [31,61]. In addition, recent cryoEM and cryoET models of yeast NPCs suggest that NPCs are intrinsically flexible [71]. Similarly, human NPC models using artificial intelligence-based structure prediction and X-ray crystallography and single-particle cryoEM structures also suggest that NPCs are highly dynamic with the ability to dilate and constrict the central NPC channel [72,73]. These observations support a model in which NPCs dilate to facilitate the transport of particularly large cargos, such as HIV capsids. Despite this, recent super-resolution correlative microscopy and cryoET studies supported by computational simulations suggest that capsid translocation may somehow break NPC rings, visualized through the loss of NPC symmetry in primary MDMs [31]. The implications of HIV transport-breaking NPCs remain unclear.

How HIV capsids actually cross the NPC has been an important question. The NPC is a highly selective and regulated gateway into the nucleus, built of multiple copies of around 30 different ‘nups’. The central channel is an intrinsically disordered, phase-separated, mesh-like permeability barrier made of repeating FG domains from FG-bearing nups [74,75]. Nuclear transport receptors, such as karyopherins, phase separate into the FG mesh and carry their cargos across the NPC [76]. Early data suggested that HIV capsids uncoated in the cytoplasm to reveal a pre-integration complex (PIC) comprising integrase, viral genome and possibly some remaining capsid protein [13]. In this model, PICs must be transported through the nuclear pore by a nuclear transport receptor. The transporter transportin-3 (TNPO3), originally a yeast-2-hybrid hit as an integrase binder, was described as a cofactor for HIV-1 infection because its depletion appeared to prevent HIV-1 nuclear transport [77,79]. This was reasonably interpreted as evidence for a role for TNPO3 in HIV nuclear import [77,79]. However, later elegant work demonstrated that TNPO3 in fact transports the HIV cofactor CPSF6 into the nucleus. In the absence of TNPO3, cytoplasmic CPSF6 meets HIV too early, blocking capsid nuclear transport [80,81].

Recent studies provide further functional details of HIV nuclear transport, showing that HIV capsids traverse the NPC by phase separating into the FG permeability barrier in much the same way as karyopherins do – via FG recruitment [82,83]. The HIV capsid does not need a karyopherin; it is a karyopherin. The CA hydrophobic FG-binding pocket, CA N57 particularly, interacts with Sec24C, Nup153 and CPSF6, as well as FG-containing nups in the NPC including Nup58, Pom121, Nup214, Nup62, Nup42 and Nup98 (Box 1). Nup98 is key because it contributes the most FGs to the permeability barrier. The fact that N57 is crucial for NPC FG interactions explains why N57 mutations, but not mutations which prevent interactions with other FG cofactors (e.g. N74D and A77V), reduce HIV infectivity of non-dividing cells [14,80, 82, 84,86]. Over 1200 FG-binding sites in an HIV capsid provide specific, yet relatively weak NPC interactions, facilitating NPC passage [82].

Cofactors such as Nup358, which bind capsid in the cytoplasm and outer nuclear envelope, may help ‘feed’ and orientate capsids into the nuclear pore via competitive interactions for the CypA loop and FG-binding sites. Nuclear transport may be promoted by the capsid’s cone shape. It is possible that subtle differences between binding affinities across the lattice could drive directionality and orientation. Capsids appear to enter the nuclear pore narrow end first, and the number of FG-binding sites increases towards the broad end; thus, an avidity gradient may be generated to facilitate directionality [87]. Experimentally, CA hexamers and capsid-like particles can enter condensates formed from recombinant Nup98 protein, and treatment with a CPSF6_313-327_ peptide removes them, presumably by competing for CA FG-binding sites, supporting a role for CPSF6 in ‘pulling’ capsids through nuclear pores [82]. Indeed, CA mutations, which prevent CPSF6 binding (A77V or N74D) or CPSF6 depletion, lead to capsid accumulation at the nuclear envelope and retargeting of integration to chromatin close to the NPC [5,62, 67,69, 88, 89].

## CPSF6 in capsid uncoating

Though it is now broadly accepted that HIV capsid uncoating occurs in the nucleus, close to, if not in association with target chromatin, the precise molecular uncoating trigger remains unclear. Here, we discuss the current evidence for the regulation of uncoating and suggest a model in which cofactors collectively regulate capsid stability through dynamic allostery.

Small molecules, which bind capsid at the FG site, including experimental capsid inhibitor PF74 and first-in-class capsid targeting drug LEN, cause capsid uncoating [90,92]. They flatten the capsid lattice, restricting its flexibility, leading to fracturing [92]. Single-molecule fluorescence experiments have shown that whilst PF74 and LEN accelerate initial capsid rupture, large sections of hyperstable lattice remain after initial fracturing [90,91]. Similarly, in the absence of inhibitors, capsids do not appear to uncoat in a single disassembly event in the nucleus; rather, they lose chunks of lattice [70,92]. These are hints that natural HIV uncoating may follow a similar process with authentic uncoating in the nucleus regulated by cofactors. The lattice flattening effect of PF74 and LEN has been attributed to the fact that they bridge two monomers, which is also a characteristic of FG cofactors [91,93]. CPSF6, as the only nuclear FG cofactor is an obvious uncoating regulator candidate. Indeed, CPSF6 peptide promotes capsid opening by stabilizing the lattice, although with reduced kinetics compared to LEN [91]. Furthermore, CPSF6 lacking a nuclear localization signal localizes to the cytoplasm and restricts infection, in some experiments, through causing cytoplasmic uncoating, although inhibition by competing for Sec24C and NPC FG binding also likely has a role [80,94]. In addition, live cell microscopy shows that GFP-tagged CPSF6 forms higher order complexes upon HIV-1 infection which colocalize with capsid in the perinuclear region (as well as the nucleus) and traffic on microtubules, potentially promoting nuclear import [95]. Full-length MBP-tagged CPSF6 forms oligomers and binds and disrupts tubular capsid assemblies *in vitro*, also supporting a role for CPSF6 in capsid uncoating [95]. This work also suggests, supported by a recent preprint, that CypA competitively limits CPSF6 complexes binding to capsid in the cell periphery, which may prevent CPSF6 prematurely uncoating HIV capsids [95,96].

However, capsids which cannot bind CPSF6 still uncoat, but in this case at the nuclear envelope, whilst halfway through NPCs [62,67,69, 89]. CPSF6-derived peptide binding does not cause a significant conformational change in CA hexamers that might explain the uncoating mechanism [97,98]. The recent evidence for phase-separated, biomolecular condensates containing CPSF6 and capsid, discussed in Box 1, and their apparent importance in infection is intriguing and suggests HIV as an excellent model to study nuclear phase separation and its biological functions [63,99,101]. One possibility is that the CPSF6 condensates containing HIV capsids fuse with similar condensates associated with chromatin to facilitate targeted integration. Thus, recruitment into CPSF6 phase-separated speckles may both target integration [4,5] and protect the virus from innate immune sensing [14], with a possible additional role in uncoating.

## DNA synthesis in capsid uncoating

There is mounting evidence that the completion of DNA synthesis inside the capsid also contributes to uncoating [68,70, 92, 102,106]. Whilst it was initially thought that viral DNA synthesis completes in the cytoplasm, recent work suggests that it can complete in the nucleus, consistent with the completion of RT driving nuclear uncoating [63,65, 68, 107]. Nuclear uncoating occurs roughly 10 h after RT initiation and 3 h after RT completion, and importantly, uncoating is delayed by the inhibition of RT with nevirapine [68,102, 103, 108]. Uncoating appears to occur in a two-step process; initial integrity loss is followed by complete lattice disassembly, and DNA synthesis only promotes the former step [103]. It has been proposed that uncoating is driven by dsDNA formation because it takes up more space than the ssRNA genome and therefore exerts mechanical force on the capsid to drive uncoating [109]. Indeed, AFM experiments on isolated HIV-1 capsids show that dsDNA formation increases pressure inside the capsid, increasing its ‘stiffness’ or ‘brittleness’ [104]. Capsids appeared to swell and rupture at the narrow end of the cone before full disassembly, whilst capsids remained intact in the absence of RT [104]. Subsequent work showed that different RT steps correlate with ‘stiffness’ spikes and changes in capsid morphology [106]. This model is also supported by the observation that uncoating efficiency correlates with genome size, with longer genomes driving more efficient uncoating than shorter genomes [102]. In addition, *in vitro* endogenous RT experiments using capsids isolated from virions showed that newly formed DNA can protrude out from capsid openings at RT completion, potentially explaining the trigger for uncoating [92]. Together, these data suggest an optimal genome size for uncoating. This model may be important for lentivector gene therapy, in which genome size and sequence are altered by vector design and transgene incorporation, potentially influencing uncoating and therefore lentivector infection efficiency. Understanding how uncoating is regulated by genome synthesis thus has the potential to improve lentivector design.

## IP6 in capsid uncoating

IP6 is expected to have a role in the regulation of capsid stability and uncoating. The IP6 concentration in cells is ~40 µM [92], yet much higher concentrations (0.2–4 mM) are needed for *in vitro* capsid formation [110,111]. Thus, IP6 is concentrated in the virion through interactions with Gag to drive mature CA assembly [111,113]. However, IP6 may be lost from capsids on entering the lower cytoplasmic IP6 concentration, which would likely have a destabilizing effect. Perhaps as IP6 is lost, cofactor regulation of capsid stability becomes increasingly important. For example, the binding of FEZ1, which also interacts with the R18 pore, may displace IP6 and stabilize capsid [52].

## A model for cofactor regulation of capsid stability by dynamic allostery

We hypothesize a model in which capsid stability is regulated by multiple, sequential cofactor interactions. We envisage cofactor-driven changes in dynamic allostery with each interaction determining the impact of the next in a stepwise directional regulation of capsid stability. Whilst the structures of capsid cofactor complexes evidence little conformational change on cofactor recruitment, evidence for dynamic allostery comes from magic angle spinning nuclear magnetic resonance. In these experiments, the assembled capsid is highly dynamic, particularly at its loops as expected. Of course, CypA-binding loop dynamics are reduced by CypA recruitment, but intriguingly, CypA binding alters dynamics across the CA protein. Critically, this included changes in dynamics at N57, the key residue in the FG-binding site [114]. Similarly, molecular dynamic simulations highlight an allosteric network of residues linking the CypA-binding loop with the CA hinge region that connects the NTD and CTD, which is influenced by activity at the FG-binding site. Specifically, the CypA loop is dynamically coupled to the CTD via *α*-helix 7 [115]. Furthermore, in the presence of inhibitor PF74, the dynamic connection occurs via an alternative pathway involving *α*-helix 4, resulting in a stronger coupling and of course reduced infection [115]. This suggests that the FG-binding site and the CypA-binding loop are allosterically connected and that the inhibitory mechanism of PF74 may involve breaking this dynamic allostery. These data may also explain how CypA promotes PF74 antiviral activity and suggest why CypA loop mutants such as H87P can contribute to PF74 resistance by re-establishing the allostery without preventing PF74 binding [115,117]. CypA loop mutations H87P and P90A also decreased the potency of FG site targeting capsid inhibitor GSK878, consistent with this model [118].

Molecular frustration theory considers how energy is distributed in protein structures and can be used to infer dynamics [119,121]. Regions that can adopt multiple conformations, or tolerate mutation without destabilizing the protein, are described as frustrated and are likely to be dynamic. These residues are typically on the protein surface or loops and represent binding or allosteric sites. Regions that are minimally frustrated are likely to be more stable and unlikely to move or mutate. We recently suggested that CypA allosterically alters CA molecular frustration distantly from its binding site, adding to the evidence for a dynamic allostery mechanism in which each cofactor recruitment prepares the capsid state for the next interaction to allow sequential regulation of stability and provide directionality [122]. This model is supported by the observation that preventing CypA binding by mutation or inhibition makes HIV less sensitive to depletion of later cofactors, for example, Nup153 and Nup358, and alters later events such as integration site selection [4,80]. Regulating capsid state by dynamic allostery across cofactor binding sites could regulate uncoating to ensure it occurs at exactly the right time and location, that is, once RT has completed, in the nucleus, and next to chromatin of the preferred nature. Interactions between capsid and viral DNA upon completion of RT may be a final signal for uncoating, opening the viral capsid just prior to integration, thereby avoiding cGAS detection. In this model, correct integration targeting is dictated by the whole series of cofactors recruiting in the right locations and order, and this is consistent with the data [4,5, 80]. Thus, these cofactors cooperatively help to maximize infection and minimize innate immune sensing. Further investigation of the effect of full-length cofactors and DNA on capsid dynamics, necessarily in authentic viral capsids, will be required to test this model [123]. Cofactors that bind capsid and their currently known functions are summarized in Table 1.

**Table 1.** Capsid-binding host factors which regulate HIV-1 infectivity

Cofactor	Cellular role	Cellular location	Key residues for binding	Function in HIV infection	Reference
Sec24C	Component of COPII coat complex	Cytoplasm	N57	Unclear, likely promotes capsid function including nuclear import	[141]
CypA	Peptidyl-prolyl isomerase enzyme	Cytoplasm and possibly nucleus	G89, P90, P123, I124, P125	Stabilizes capsid and protects from TRIM5*α*	[28,30, 220,225]
TRIM5*α*	IFN-induced viral restriction factor	Cytoplasm	Capsid lattice and curvature	Restricts infection by caging viral capsids and preventing nuclear transport as well as stimulating innate immune activation and pulling viral capsids apart in association with proteasomes	[41,42, 44,47]
IP6	Small molecule metabolite	Throughout cells	R18, K25	Promotes assembly and stability of capsid lattices and therefore promotes DNA synthesis	[112,123, 240,243]
FEZ1	Kinesin-1 adaptor protein	Cytoplasm (microtubules)	R18	Capsid trafficking through cytoplasm to nuclear pore along microtubules	[52,53]
BICD2	Dynein adaptor protein and activator	Cytoplasm (microtubules)	Unknown	Capsid trafficking through cytoplasm to nuclear pore along microtubules	[54,55]
Mx2/MxB	IFN-induced GTPase	Outer nuclear membrane, near nuclear pores	E75, E212, E213	Restricts infection by interfering with nuclear entry	[250]
Nup358 (RanBP2)	Nup	Outer nuclear pore/microtubules	G89, P90	Capsid nuclear entry and integration site targeting	[4,56, 228, 229]
Nup58, Pom121, Nup214, Nup62, Nup42 and Nup98	Nups	NPC	N57	Import of capsid through the nuclear pore as a karyopherin	[82,83]
Nup153	Nup	Inner nuclear pore	N57, Q63, E75, R143, R173, Q176, A177, E212, E213	Nuclear import of capsid	[80,84, 93, 249]
CPSF6	Component of CFIm complex involved in pre-mRNA processing	Nucleus	N53, L56, N57, M66, Q67, K70, I73, N74, S109, T107, A105, Y130, Q179, K182	Extraction of capsids from the nuclear pore, trafficking of capsid to integration sites in chromatin, likely through phase separation, maybe uncoating	[4,80, 93, 98]

## Do capsid adaptations influence innate immune activation, transmission rates and pathogenesis?

The zoonotic origin of HIV is well understood, with 13 HIV zoonoses in humans detected to date: HIV-1(M) and HIV-1(N) from chimpanzees, HIV-1(O) and HIV-1(P) from gorillas and nine distinct HIV-2(A–I) from sooty mangabeys. However, they are not equal in their human transmission. Most are very rare; for example, HIV-1(N) and (P) and HIV-2(C–I) have only been detected once, twice or a handful of times [124]. Only HIV-1(M) is pandemic, accounting for over 95% of 86 million infections, and HIV-2(A) is the majority of the remainder at around 1.5 million cases [125]. This puts HIV-1(M) into a pandemic class of its own. Without this single zoonotic event, HIV infection would be very rare and largely restricted to West Africa, and given that HIV-2 infection is less frequently associated with immune deficiency [126,128], HIV would rarely be associated with AIDS. What determines different HIV transmission rates and pathogenesis? Studies in macaques experimentally infected with sooty mangabey-derived SIVmac and treated with IFN-I inhibitors, or IFN-I itself, demonstrate that timing is everything. Early IFN-I reduced the success of experimental challenge and improved viral control and thus symptoms [129]. Late IFN-I, however, worsened the disease. Here, we make the case that, because it is a central influencer of innate immune activation [20,22,130, 131], and this distinguishes pandemic HIV-1(M) from non-pandemic HIV-1(O) and HIV-2 [132], capsid is a central determinant of transmission, pathogenesis and pandemic potential.

Whilst both HIV-1 and HIV-2 use the same receptors for cell entry (CD4 and CXCR4 or CCR5, although HIV-2 use of coreceptors is more promiscuous), only HIV-1 replicates efficiently in macrophages [132,137]. Although HIV-2 is less well characterized than HIV-1, evidence suggests that they use a similar set of cofactors. The FG-binding site is conserved [98], and both viruses can infect non-dividing cells [138], suggesting conserved functional interactions with FG nups. Indeed, HIV-2 appears to also be dependent on Nup153 and Nup358 for infection [139] and is sensitive to restriction by TRIM-Nup153_C_ (CTD) [84]. As with HIV-1, CPSF6_313-327_ peptide binds HIV-2 NTD [98], and HIV-2 is restricted by cytoplasmic truncated CPSF6, which has lost its nuclear localization signal (CPSF6-358) [80]. Indeed, CPSF6 and LEDGF/p75 are also important for targeting HIV-2 integration, as they are for HIV-1, with HIV-2 also favouring integration into transcriptionally active SPADs and disfavouring heterochromatic lamina associated domains [2,88, 100, 140]. The Sec24C interaction also appears to be conserved across primate lentiviruses, as TRIM-Sec24C_196-314_ restricts SIVmac239, SIVstm and SIVsmE041 and Sec24C KO reduced infectivity of these viruses [141].

HIV-2 isolates tend not to depend on CypA but can be restricted by certain TRIM5-CypA alleles, suggesting CypA recruitment to capsid [142,143]. HIV-1 is not typically sensed by cGAS/STING as described in previous sections, but HIV-2 viral cDNA can be sensed in the cytoplasm [130,132]. CypA seems to have a greater role in regulating the viral capsid stability of HIV-1 than HIV-2. In one study, transferring the CypA-binding loop from HIV-1 to HIV-2 increased cGAS-mediated HIV-2 sensing [132]. This was interpreted as CypA recruitment contributing to sensing, but we interpret this result as showing that mutating the CypA-binding loop, in either virus, disturbs the protection of viral DNA by capsid, leading to the detection by cGAS. For example, the HIV-1 cyclophilin-binding mutant P90A triggers IFN production in macrophages in a DNA synthesis-dependent manner although TRIM5 also has an important role in this process [14,28].

Phylogenetic and structural comparisons of HIV-1(M) with HIV-1(O) and HIV-2 have suggested specific capsid adaptations, made by the chimpanzee parent of HIV-1(M), that are uniquely associated with the pandemic lineage. Firstly, pandemic and non-pandemic HIV-1 CA hexamer structures are quite different from each other. Pandemic HIV-1(M) hexamers can be solved by X-ray crystallography in two conformations with the beta hairpin (BHP) open or closed over the central electrostatic pore [132,144]. However, non-pandemic HIV-1(O) can only be solved in an open position. Critically, phylogenetic comparisons suggest why. The pandemic HIV-1(M) lineage has gained glutamine in place of the ancestral tyrosine at position CA 50. Q50 in pandemic HIV-1(M) allows the coordination of a water molecule that pushes the BHP into a closed position. With a tyrosine at this position, HIV-1(O) cannot form the closed pore position. HIV-2 CA also bears tyrosine at this position, and although an HIV-2 CA hexamer structure is lacking, HIV-2 hexamers are expected to form an open conformation. In addition, pandemic HIV-1(M) has lost an arginine at position CA 120 (R120). In HIV-1(O), this residue forms a salt bridge with E98 at the base of the CypA-binding loop, whilst in HIV-2, the equivalent residue (R118) forms a salt bridge with E96 [132]. These observations suggest that the CypA-binding loop and BHP have evolved to be more dynamic in pandemic HIV-1(M).

**Fig. 4.**
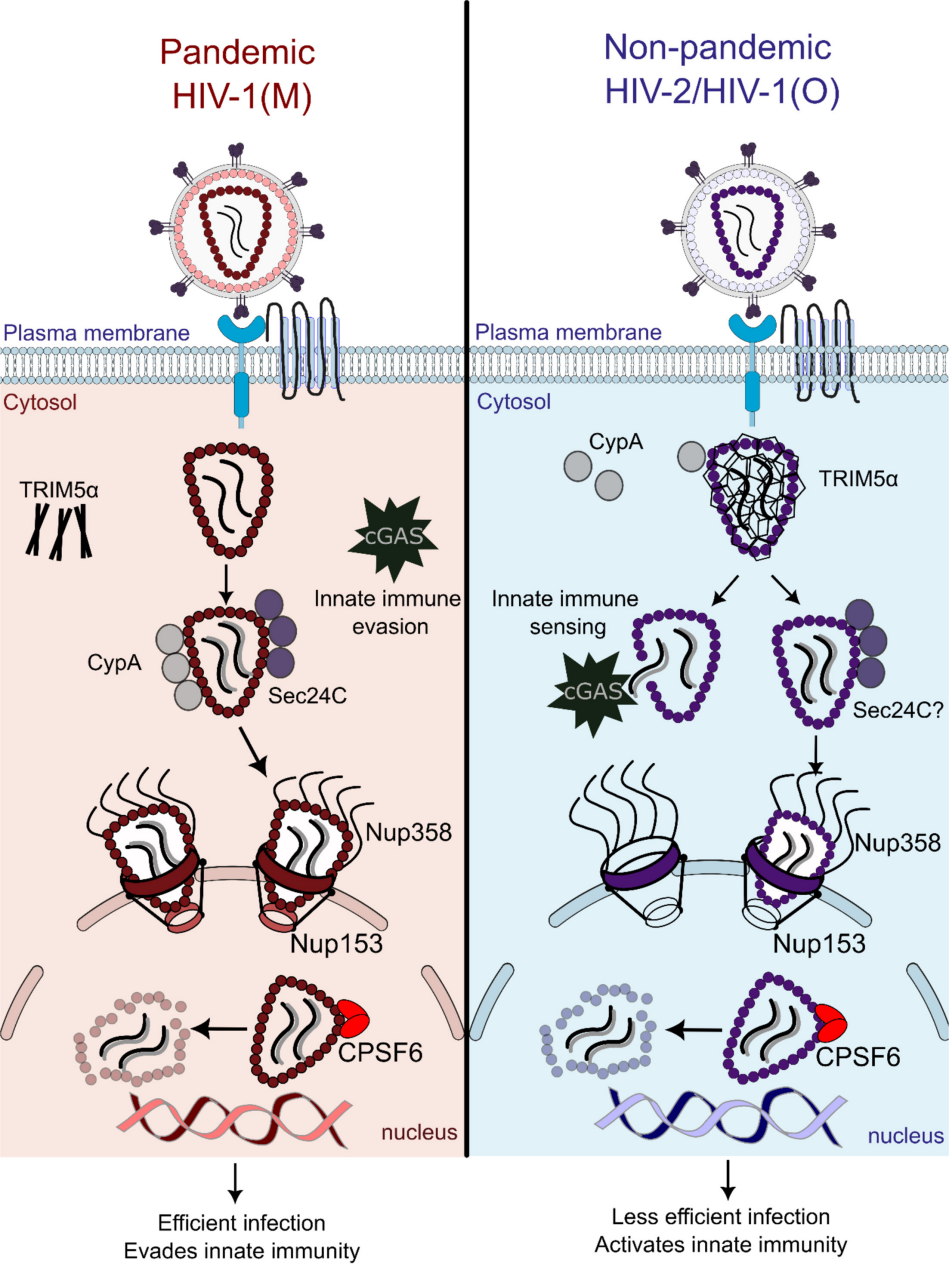
HIV-1 capsid has evolved to evade innate immunity. Pandemic HIV-1(M) capsid is better able to shield viral DNA from nucleic acid sensor cGAS, enabling RT to take place undetected [132]. Similarly, it is able to better escape TRIM5*α* restriction, compared to non-pandemic HIV-1(O) and HIV-2. These adaptations enable HIV-1(M) to replicate better in myeloid cell models and may promote human-to-human transmission [132]. Whilst interactions with the FG cofactors appear to be conserved, HIV-1(M) appears to bind more tightly to CypA and is more dependent on it for infection [28,145].

We argue that increased dynamics could make the capsid more regulatable, particularly through dynamic allostery driven by cofactor binding. This model is supported by the pandemic virus, with its particularly mobile CypA-binding loop, being most dependent on CypA binding for the regulation of capsid stability and infectivity. Importantly, non-pandemic HIV-1(O) and HIV-2 are less dependent on CypA and more effectively trigger innate immune signalling by TRIM5*α* and cGAS, dependent on capsid. Mutating pandemic HIV-1(M) to reverse capsid adaptations (Q50Y+R120) produced a virus with an open central hexamer pore, which activated cGAS and TRIM5*α* innate immune sensing to a similar level as the non-pandemic viruses [132]. Interestingly, non-pandemic HIV-1(N) and HIV-1(P) are also more sensitive to human TRIM5*α*, and CypA does not seem to protect any of the non-pandemic viruses from TRIM5*α* in the same way as it does for pandemic HIV-1(M) [28,132, 145] (Fig. 4). Over-expression of TREX1 abolished the activation of cGAS, but not TRIM5*α*, by non-pandemic capsids, consistent with non-pandemic HIV exposing viral cDNA inappropriately. These pandemic-associated adaptations enable HIV-1(M) to replicate efficiently in myeloid cell models, whereas HIV-1(O) and HIV-2 activated IFN production, which suppressed the replication. Although comparison of innate immune activation by HIV is typically performed in myeloid cells, because they most effectively respond to HIV infection, it is certainly possible that the transmission advantage from enhanced innate immune evasion derives from more efficient replication in T-cells *in vivo* through minimal induction of the IFN-I responses that have been shown to suppress lentiviral transmission [129]. Thus, capsid adaptations contribute to effective innate immune evasion, enabling more elective transmission and contributing to pandemicity.

## Vpr and Vpx dictate an infection niche for immunodeficiency viruses

In addition to avoiding innate immune sensing of viral nucleic acids by hiding genome within capsids, lentiviruses encode Vpr and Vpx proteins that manipulate innate immunity and infected cell biology. Both HIV-1 and HIV-2 capsids encapsidate accessory protein Vpr, but HIV-2 and its sooty mangabey-derived parental lineage additionally encode and encapsidate a Vpx protein. Vpr and Vpx are related to 12–16 KDa proteins of around 100 aas. They fold into three-helix bundles with long disordered N- and C-termini [146,148].

During viral assembly, Vpr and Vpx are recruited through interaction with the p6 region of Gag making them the only accessory proteins packaged in abundance into virions [149], highlighting their importance in the early stages of HIV infection. Given that not all the CA protein is used to form the capsid, some Vpr/Vpx will inevitably lie between the capsid and the viral membrane and be released directly into the cytoplasm on fusion. Whether this protein has a role in infection is unclear but we certainly expect it to. Localization studies indicate that Vpr and Vpx quickly traverse from the cytoplasm to the nucleus during infection and when overexpressed [150,151].

Vpr/Vpx similarity suggests that they are derived from one or more gene duplication events followed by divergence [152,153], but divergence and functional overlap prevent a clear view of ancestry. Typically, if a virus carries two, the second one is called Vpx, and if there is only one, it is called Vpr. Functionally, both proteins share a conserved association with the DDB1-cullin-RING E3 ubiquitin ligase, via the DCAF-1 adaptor protein, which allows them to target host proteins for ubiquitination and proteasomal degradation [154]. In this section, we focus on the role of Vpr and Vpx in defining viral tropism through regulating infected cell biology and innate immune sensing.

Despite a degree of functional overlap, Vpr and Vpx diverge in their targets in a lineage-dependent manner [155,157]. For example, HIV-2 Vpx, but not Vpr of most immunodeficiency virus lineages, targets SAMHD1 for degradation via the E3 ubiquitin ligase [158,159]. SAMHD1 acts as a restriction factor by regulating the production of nucleotides essential for viral genome synthesis [160,161]. SAMHD1 is regulated during cell division. In macrophages or dendritic cells, which differentiate out of cell cycle into G0, cyclin A2/cyclin dependent kinase 1 (CDK1) phosphorylates SAMHD1 at T592 activating it, reducing nt levels and limiting viral genome synthesis [162,165]. Dephosphorylation at T592 switches SAMHD1 off, allowing nucleoside triphosphate production [165] and promoting virus replication. One might therefore expect that encoding a SAMHD1 antagonist in Vpx would allow HIV-2 uninterrupted replication in macrophages and dendritic cells. Indeed, HIV-2 is more infectious than HIV-1 in single-round infections on these cells, and this is dependent on Vpx [166]. Furthermore, mutating HIV-1 p6 so that it can incorporate Vpx expressed in trans [167] and treating cells with virus-like particles (VLPs) bearing Vpx both enhance HIV-1 single-round infection in myeloid cells [131,166, 168, 169]. In these experiments, VLPs are typically made by transfecting VSV-G envelope and Gag-Pol bearing packaging construct, but no genome plasmid. These observations are consistent with Vpx degradation of SAMHD1, providing an infection advantage for Vpx-bearing viruses such as HIV-2 in myeloid cells. However, Vpx-bearing viruses do not replicate in myeloid cells *in vitro* [132,133, 170, 171], and whilst HIV-2 infects dendritic cells (DC) and macrophages, it tends to trigger innate immune sensing via cGAS, leading to the suppression of replication [130,132,170, 171]. Thus, enhancing RT by Vpx-mediated SAMHD1 degradation is a double-edged sword and is not associated with efficient replication in myeloid cells. How does HIV-1 infect myeloid cells without suppressing SAMHD1? By studying HIV-1 replication in MDM, Mlcochova *et al*. discovered that MDM cycle in and out of a G1-like state that is marked by elevated MCM (minichromosome maintenance protein) and CDK1, which phosphorylates and deactivates SAMHD1, allowing for HIV-1 replication without Vpx [162]. We propose that lacking Vpx, and bearing a capsid adapted to precisely uncoat just before integration [132], defines a niche in which HIV-1 can replicate in CD4+ T-cells and macrophages, whereas HIV-2 is restricted to CD4+ T-cells due to the enhanced sensing and antiviral state it drives in macrophages. Consistent with this model, Vpx has been shown to enhance infection of T-cells, particularly if they are in the resting state [172]. Importantly, Vpx-bearing viruses are not found infecting macrophages *in vivo* in their natural hosts [173].

## Vpx targets HUSH for degradation

The HUSH complex was discovered as a key regulator of HIV-1 expression in 2015. In this elegant study, Tchasovinkarova *et al*. discovered that HUSH suppressed GFP expression from an HIV-1-derived vector [174]. HUSH, comprising transgene activation suppressor (TASOR; also known as FAM208A), M-phase phosphoprotein 8 (MPP8) and Periphilin (PPHLN1), is an epigenetic regulator complex that regulates the expression of newly integrated elements such as retroviruses as well as mobile elements in the human genome. How target selectivity works is poorly understood, but recent models propose that PPHLN1 recognizes long intronless transcripts, for example, LINE-1, or newly integrated retroviruses, which allows it to distinguish between classical gene transcripts and intronless transcripts that are seen as harmful [175]. MPP8 is recruited to genomic areas rich in repressive H3K9me3 via the chromodomain of MPP8. SETDB1 recruitment then deposits further repressive histone marks [174,176, 177]. HUSH also recruits chromatin remodeller MORC2 to compact chromatin and regulates RNA expression post-transcriptionally by interacting with the degradative nuclear exosome targeting complex [178]. In the context of HIV, TASOR silences provirus expression by interacting with mediators of RNA metabolism such as CNOT1, RNA polymerase II and RNA exosomes to degrade viral RNA during and after transcription [179]. Thus, HUSH is able to regulate the transcription of host genes, type 1 mobile elements and viruses using complex mechanisms.

Prior to the detailed study of HUSH mechanisms, back-to-back studies identified Vpx as an antagonist of HUSH-mediated lentiviral gene repression [168,180]. Vpx targets TASOR, and to a lesser extent MPP8, for degradation dependent on DCAF-1 and the CUL4A/B E3 ubiquitin ligase complex. Degradation is rapid, starting as early as 30 min after infection, and depletion of one HUSH component tends to result in loss of others [168,180]. HUSH degradation is genetically separable from SAMHD1 degradation, as shown by Vpx mutant Q47A V48A, which degrades SAMHD1, but not TASOR. Consistent with a viral repression function for HUSH, its degradation by Vpx enhanced the replication of HIV-1 in Jurkats and SIVmac in CEMx174 cells [168,180]. Additionally, delivery of Vpx to HIV-1 latency model T-cell line JLat reduces H3K9me3 chromatin modifications and activates expression from the latent provirus, particularly in the presence of Tumour Necrosis Factor (TNF), which stimulates the viral promoter [168,180]. However, as with SAMHD1, HUSH deregulation may also be a double-edged sword because it may activate innate immunity by inducing the expression of transposable element-related sequences including LINE1 and endogenous retroviruses, which are otherwise silenced by HUSH [175,181]. Intriguingly, a recent study demonstrated the importance of TASOR2, a TASOR paralogue which forms a HUSH2 complex with MPP8 and PPHLN1. This complex has activity against ISG expression, suggesting that Vpx may distinguish between HUSH and HUSH2 to activate viral gene expression whilst continuing to repress ISGs [182].

## The enigma of Vpr: enhancing proviral expression but evading innate immune activation

A clear model of how Vpr enhances lentiviral replication is elusive. Vpr is certainly required for replication *in vivo*. A SIVmac mutant lacking Vpr displayed delayed replication kinetics, rapid innate immune activation and stronger B- and T-cell responses in rhesus macaques, leading to better preservation of CD4+ T-cells, lower viral loads and an attenuated clinical course [183]. Furthermore, accidental infection of a lab worker with an HIV-1 lacking Vpr did not lead to AIDS [184]. However, replication defects for HIV-1 Vpr mutants *in vitro* are very context dependent. Vpr is typically not required for HIV-1 replication in cell lines or even reliably in primary T-cells and macrophages. There are examples in which Vpr mutation has caused defective replication, but the experimental differences which underlie this requirement are typically unclear [185,190]. In one study, treating MDMs with cGAS product cGAMP rendered HIV-1 replication Vpr dependent, consistent with a role for Vpr in innate immune evasion [191]. Certainly, Vpr causes huge DCAF1-dependent changes in the infected T-cell proteome [155], which are likely influenced by Vpr regulation of the infected cell transcriptome as well as by direct degradation of Vpr target proteins [192]. Vpr targets include DNA damage repair and cell cycle progression regulators uracil-DNA glycosylase-2 (UNG2), MUS81 and EME1, and the modified transcriptome has been linked to driving T-cell residency [155,192].

The Vpr C-terminal domain interacts with karyopherins and regulates nuclear transport [191,193]. Overexpressing Vpr, or delivering it via VLP, dampened ISG induced by various pathogen-associated molecular patterns including cGAMP, HT-DNA or poly(I:C), in the monocytic cell line THP-1 and primary human MDM. Vpr suppressed the phosphorylation of interferon regulatory factor 3 (IRF3) at S396 but not S386 and reduced IRF3 and NFκB nuclear localization dependent on Vpr residues F34/P35 [191]. In this study, KPNA1 interaction was dependent on Vpr F34/P35. The authors concluded that Vpr inhibits activated transcription factor nuclear transport by targeting karyopherins to suppress innate immune responses [191]. Vpr manipulation of karyopherins is supported by an X-ray crystal structure of a Vpr-derived peptide, residues 85–96, in complex with importin-*α* (KPNA1) [148]. Note that the peptide used in this study does not correspond to the Vpr F34/P35 mutant used in [191], suggesting a complex interaction. Notably, HIV capsids are hypothesized to traverse nuclear pores in a karyopherin-independent manner, likely explaining why Vpr does not inhibit HIV-1 nuclear transport [82,83].

Interestingly, whilst HIV-1 Vpr has been shown to promote proviral expression and reactivate latently infected cells, it does not appear to degrade HUSH [12,155, 180]. HIV-1 Vpr mediates the degradation of histone deacetylases (HDAC1 and HDAC3) alongside the heterochromatin modulator CTIP2, allowing for the recruitment of NFκB subunit, p65 and the transcription of HIV-1 LTR [12,194]. Although HIV-1 enhances LINE-1 transcription, overexpressing HIV-1 Vpr on its own typically does not [195]. In fact, Vpr typically prevents LINE-1 retrotransposition in a dose-dependent manner [195]. Although Vpr modulates epigenetic regulators, its effect on transposable element expression and how this influences innate immune activation is understudied.

When HIV capsids uncoat in the nucleus, if the viral DNA fails to immediately integrate, the LTRs on either end of the reverse-transcribed genome can either be ligated, by the host cell machinery into structures known as 2-LTR circles, or form 1-LTR circles through recombination [196,197]. These extrachromosomal HIV DNA circles mark nuclear entry because the enzymes required for their formation reside in the nucleus. They are thought to be uninfectious but can accumulate, particularly in patients taking integrase inhibitors [198,199]. Recent studies asked why these circular HIV genome forms do not lead to the production of infectious viruses. They found that SMC5–SMC6 localization factor 2 (SLF2) targets SMC5/6 to unintegrated viral DNA, leading to topological entrapment and depletion of active transcription histone modifications, H3K4me3 and H3K9ac independently of HUSH [200,201]. However, Vpr-mediated proteasomal degradation of SLF2 relieves this repression and may allow circles to contribute to viral gene expression [200]. In this way, Vpr may avoid enhancing transposable element expression and innate immune activation by acting on unintegrated DNA circles, rather than integrated proviruses, which may be difficult to distinguish from other integrated virus-like sequences. Notably, SLF2 degradation is conserved across diverse lentiviral lineages, indicating its importance to lentiviral replication.

Many studies have linked Vpr to DNA damage and cell cycle arrest [202,206] with some associating these phenotypes with innate immune evasion [191,202,204]. Vpr is hypothesized to induce DNA damage by associating with the SLX4 complex, which normally resolves aberrant DNA intermediates such as Holliday junctions to protect genomic integrity and resume the cell cycle [207]. Vpr-mediated ubiquitination of SLX4 complex component MUS81 causes its untimely activation, leading to DNA double-strand breaks, FANCD2 foci and cell cycle arrest [204,208]. One possibility is that SLX4 complex activation leads to the degradation of viral reverse transcripts that would otherwise activate innate immune sensing [191,202,204]. Vpr mutation R80A failed to interact with Mus81 and did not induce DNA damage or cell cycle arrest. Vpr DCAF-1-binding domain, Q65R, also lost this activity, but this may be explained by Vpr mutant mislocalization, reported in various cell lines [191]. In another study, Vpr degradation of MUS81 was confirmed and extended to the post-replication DNA repair helicase HLTF. Thus, given that Vpr also degrades UNG2, Vpr manipulates proteins in three independent DNA repair pathways [209]. Finally, Vpr degradation of CCD137 was linked to cell cycle arrest and enhancement of HIV-1 gene expression [206]. Thus, Vpr has a complex impact on host cell biology, also reflected in proteomic studies, demonstrating that Vpr can manipulate at least 38 different cellular targets, eventually leading to huge changes in the infected cell proteome [155].

Box 2: **Thoughts on experimental methods**

MDMs and monocyte-derived dendritic cells (MDDCs) have been used extensively as cell models for HIV infections [100,130,132]. Both cell models are differentiated from isolated PBMCs in the presence of different cytokines. MDDCs are typically differentiated in the presence of 10 ng ml^−1^ recombinant human granulocyte-macrophage colony-stimulating factor (GM-CSF) and 50 ng ml^−1^ IL-4 and MDMs using 100 ng ml^−1^ macrophage colony-stimulating factor (M-CSF). However, the literature is rich in subtly diverse isolation protocols, which may translate into significant phenotypic differences in the cells produced. For example, the isolation of monocytes prior to cytokine stimulation can be carried out using selection by adherence to plasticware [132], or purification with CD14+ beads before stimulation with M-CSF [131]. Growth in autologous human serum, pooled human serum or FCS all have a significant impact on cell phenotypes. For example, MDMs adopt a different cell cycle state dependent on the source of serum [257] tending to remain in G0 in human serum, but cycling between G0 and a G1-like state during which SAMHD1 is switched off in FCS [258]. We expect that such subtle differences in protocols can underlie challenges of replicating results across studies, particularly when the phenotype under study is highly dependent on the nature of the target cells, for example, demonstrating a replication defect for HIV-1 Vpr mutants. Our approach is to establish protocols that produce cells that give reliable and repeatable differential infection phenotypes, for example, comparing a WT and mutant virus [14], or HIV-1 and HIV-2 [132] and then to work out the molecular mechanisms of those differences with the expectation that such new knowledge will be informative. We argue that the discoveries we make are most relevant to the situation *in vivo* when we are studying the molecular details of specific host–virus interactions *in vitro* rather than focusing on seeking the most realistic cellular models of infection. That is, we argue that effect size and tractability trump cell model choice although we accept that the most persuasive mechanistic discoveries will always include recapitulation of key findings in primary cells.

We expect that a better understanding of HIV capsid function is going to rely on advances in structural biology, particularly cryoEM, and using these techniques in infected cells where the capsid structures and cofactor interactions are authentic. We envisage advances in understanding how capsids interact with the cytoskeleton to traffic within cells, how they traverse nuclear pores and how they regulate uncoating and integration targeting through contact with nuclear factors and chromatin. We also argue that we must go beyond simply measuring infection when studying capsid because key roles such as avoiding innate immune activation by regulating uncoating generally have little impact on the titre of an HIV-1 GFP vector infection.

## Concluding remarks

The HIV-1 capsid is a sophisticated molecular machine that has evolved to interact intimately with its host in order that the intracellular environment dictates its behaviour: remaining intact, making DNA, going through the NPC and uncoating at exactly the right time and place. In this way, capsid orchestrates the early stages of infection and, critically, regulates the timing and location of genome release and therefore genome detection by innate immune sensors. The pivotal importance of innate immune evasion in the earliest stages of infection is also highlighted by the activity of the virion-associated proteins Vpr and Vpx. We argue that these proteins also regulate the host response to infection. For example, we hypothesize that the manipulation of SAMHD1 restricts HIV-2 replication to T-cells because it activates innate immune sensing in myeloid cells through enhancing viral DNA synthesis to a point that it gets sensed by cGAS. The role of Vpx-driven HUSH degradation in activating sensing is less clear, but certainly, HUSH loss is expected to activate genomic elements that can drive nucleic acid sensing in one way or another. We hypothesize that HIV-1 does not encode Vpx because its pandemic levels of human–human transmission depend on quiet replication, achieved in part through more subtle avoidance of suppression by SAMHD1 and HUSH in macrophages and T-cells. We argue that together, these particle-associated proteins, CA, Vpr and Vpx, are niche defining. That is, through dictating the degree of innate sensing the virus experiences, they determine the host response to infection and therefore cellular tropism, pathogenesis, transmission route, transmission frequency and pandemic potential.

## Abbreviations

AFM: atomic force microscopy
BHP: beta hairpin
CDK1: cyclin-dependent kinase 1
cGAS: cyclic GMP–AMP synthase
CPSF6: cleavage and polyadenylation specificity factor 6
cryoEM: cryo electron microscopy
cryoET: cryo electron tomography
CTD: C-terminal domain
CypA: cyclophilin A
HIV: human immunodeficiency virus
HUSH: human silencing hub
IRF3: Interferon regulatory factor 3
ISG: IFN-stimulated gene
LCR: low complexity region
MCM: minichromosome maintenance protein complex
MDMs: monocyte-derived macrophages
MPP8: M-phase phosphoprotein 8
NPC: nuclear pore complex
NTD: N-terminal domain
Nup153: nucleoporin 153
Nup358: nucleoporin 358
nups: nucleoporins
PIC: pre-integration complex
PQBP1: polyglutamine-binding protein 1
RT: reverse transcription
SPADs: speckle-associated chromatin domains
STING: stimulator of IFN gene
TASOR: transgene activation suppressor
TNPO3: transportin-3
TREX1: three prime repair exonuclease 1
UNG2: uracil-DNA glycosylase-2
VLP: virus-like particle

## References

[1] Malim MH, Bieniasz PD (2012). HIV Restriction Factors and Mechanisms of Evasion. Cold Spring Harb Perspect Med.

[2] Marini B, Kertesz-Farkas A, Ali H, Lucic B, Lisek K (2015). Nuclear architecture dictates HIV-1 integration site selection. Nature.

[3] Schröder ARW, Shinn P, Chen H, Berry C, Ecker JR (2002). HIV-1 integration in the human genome favors active genes and local hotspots. Cell.

[4] Schaller T, Ocwieja KE, Rasaiyaah J, Price AJ, Brady TL (2011). HIV-1 capsid-cyclophilin interactions determine nuclear import pathway Integration Targeting and Replication Efficiency. PLoS Pathog.

[5] Sowd GA, Serrao E, Wang H, Wang W, Fadel HJ (2016). A critical role for alternative polyadenylation factor CPSF6 in targeting HIV-1 integration to transcriptionally active chromatin. Proc Natl Acad Sci U S A.

[6] Maertens G, Cherepanov P, Pluymers W, Busschots K, De Clercq E (2003). LEDGF/p75 is essential for nuclear and chromosomal targeting of HIV-1 integrase in human cells. J Biol Chem.

[7] Nikolaitchik OA, Islam S, Kitzrow JP, Duchon A, Cheng Z (2023). HIV-1 usurps transcription start site heterogeneity of host RNA polymerase II to maximize replication fitness. Proc Natl Acad Sci U S A.

[8] Friedman J, Cho W-K, Chu CK, Keedy KS, Archin NM (2011). Epigenetic silencing of HIV-1 by the histone H3 lysine 27 methyltransferase enhancer of Zeste 2. J Virol.

[9] Ruelas DS, Greene WC (2013). An integrated overview of HIV-1 latency. Cell.

[10] Chougui G, Margottin-Goguet F (2019). HUSH, a link between intrinsic immunity and HIV latency. Front Microbiol.

[11] Romani B, Allahbakhshi E (2017). Underlying mechanisms of HIV-1 latency. Virus Genes.

[12] Romani B, Kamali Jamil R, Hamidi-Fard M, Rahimi P, Momen SB (2016). HIV-1 Vpr reactivates latent HIV-1 provirus by inducing depletion of class I HDACs on chromatin. Sci Rep.

[13] Fassati A, Goff SP (2001). Characterization of intracellular reverse transcription complexes of human immunodeficiency virus type 1. J Virol.

[14] Rasaiyaah J, Tan CP, Fletcher AJ, Price AJ, Blondeau C (2013). HIV-1 evades innate immune recognition through specific cofactor recruitment. Nature.

[15] Hilditch L, Towers GJ (2014). A model for cofactor use during HIV-1 reverse transcription and nuclear entry. Curr Opin Virol.

[16] Gao D, Wu J, Wu Y-T, Du F, Aroh C (2013). Cyclic GMP-AMP synthase is an innate immune sensor of HIV and other retroviruses. Science.

[17] Sun L, Wu J, Du F, Chen X, Chen ZJ (2013). Cyclic GMP-AMP synthase is a cytosolic DNA sensor that activates the type I interferon pathway. Science.

[18] Ablasser A, Goldeck M, Cavlar T, Deimling T, Witte G (2013). cGAS produces a 2’-5’-linked cyclic dinucleotide second messenger that activates STING. Nature.

[19] Wu J, Sun L, Chen X, Du F, Shi H (2013). Cyclic GMP-AMP is an endogenous second messenger in innate immune signaling by cytosolic DNA. Science.

[20] Sumner RP, Harrison L, Touizer E, Peacock TP, Spencer M (2020). Disrupting HIV-1 capsid formation causes cGAS sensing of viral DNA. EMBO J.

[21] Eschbach JE, Puray-Chavez M, Mohammed S, Wang Q, Xia M (2024). HIV-1 capsid stability and reverse transcription are finely balanced to minimize sensing of reverse transcription products *via* the cGAS-STING pathway. mBio.

[22] Sumner RP, Blest H, Lin M, Maluquer de Motes C, Towers GJ (2024). HIV-1 with gag processing defects activates cGAS sensing. Retrovirology.

[23] Kumar S, Morrison JH, Dingli D, Poeschla E (2018). HIV-1 activation of innate immunity depends strongly on the intracellular level of TREX1 and sensing of incomplete reverse transcription product. J Virol.

[24] Papa G, Albecka A, Mallery D, Vaysburd M, Renner N (2023). IP6-stabilised HIV capsids evade cGAS/STING-mediated host immune sensing. EMBO Rep.

[25] Yan N, Regalado-Magdos AD, Stiggelbout B, Lee-Kirsch MA, Lieberman J (2010). The cytosolic exonuclease TREX1 inhibits the innate immune response to human immunodeficiency virus type 1. Nat Immunol.

[26] Davids B-O, Balasubramaniam M, Sapp N, Prakash P, Ingram S (2021). Human three prime repair exonuclease 1 promotes HIV-1 integration by preferentially degrading unprocessed viral DNA. J Virol.

[27] Prakash P, Khodke P, Balasubramaniam M, Davids B-O, Hollis T (2024). Three prime repair exonuclease 1 preferentially degrades the integration-incompetent HIV-1 DNA through favorable kinetics, thermodynamic, structural, and conformational properties. J Biol Chem.

[28] Kim K, Dauphin A, Komurlu S, McCauley SM, Yurkovetskiy L (2019). Cyclophilin A protects HIV-1 from restriction by human TRIM5α. Nat Microbiol.

[29] Selyutina A, Persaud M, Simons LM, Bulnes-Ramos A, Buffone C (2020). Cyclophilin A prevents HIV-1 restriction in lymphocytes by blocking human TRIM5α binding to the viral core. Cell Reports.

[30] Ni T, Gerard S, Zhao G, Dent K, Ning J (2020). Intrinsic curvature of the HIV-1 CA hexamer underlies capsid topology and interaction with cyclophilin A. Nat Struct Mol Biol.

[31] Kreysing JP, Heidari M, Zila V, Cruz-Leon S, Obarska-Kosinska A Passage of the HIV capsid cracks the nuclear pore. *Biophysics*.

[32] Stremlau M, Owens CM, Perron MJ, Kiessling M, Autissier P (2004). The cytoplasmic body component TRIM5alpha restricts HIV-1 infection in Old World monkeys. Nature.

[33] Wilson SJ, Webb BLJ, Ylinen LMJ, Verschoor E, Heeney JL (2008). Independent evolution of an antiviral TRIMCyp in rhesus macaques. Proc Natl Acad Sci USA.

[34] Sawyer SL, Wu LI, Emerman M, Malik HS (2005). Positive selection of primate TRIM5alpha identifies a critical species-specific retroviral restriction domain. Proc Natl Acad Sci USA.

[35] Song B, Javanbakht H, Perron M, Park DH, Stremlau M (2005). Retrovirus restriction by TRIM5α variants from Old World and New World primates. J Virol.

[36] Sayah DM, Sokolskaja E, Berthoux L, Luban J (2004). Cyclophilin A retrotransposition into TRIM5 explains owl monkey resistance to HIV-1. Nature.

[37] Stremlau M, Perron M, Welikala S, Sodroski J (2005). Species-specific variation in the B30.2(SPRY) domain of TRIM5α determines the potency of human immunodeficiency virus restriction. J Virol.

[38] Yap MW, Nisole S, Stoye JP (2005). A single amino acid change in the SPRY domain of human Trim5alpha leads to HIV-1 restriction. Curr Biol.

[39] Ohkura S, Yap MW, Sheldon T, Stoye JP (2006). All three variable regions of the TRIM5alpha B30.2 domain can contribute to the specificity of retrovirus restriction. J Virol.

[40] Maillard PV, Reynard S, Serhan F, Turelli P, Trono D (2007). Interfering residues narrow the spectrum of MLV restriction by human TRIM5alpha. PLoS Pathog.

[41] Ganser-Pornillos BK, Chandrasekaran V, Pornillos O, Sodroski JG, Sundquist WI (2011). Hexagonal assembly of a restricting TRIM5alpha protein. Proc Natl Acad Sci U S A.

[42] Li Y-L, Chandrasekaran V, Carter SD, Woodward CL, Christensen DE (2016). Primate TRIM5 proteins form hexagonal nets on HIV-1 capsids. eLife.

[43] Li X, Sodroski J (2008). The TRIM5alpha B-box 2 domain promotes cooperative binding to the retroviral capsid by mediating higher-order self-association. J Virol.

[44] Fletcher AJ, Vaysburd M, Maslen S, Zeng J, Skehel JM (2018). Trivalent RING assembly on retroviral capsids activates TRIM5 ubiquitination and innate immune signaling. Cell Host Microbe.

[45] Pertel T, Hausmann S, Morger D, Züger S, Guerra J (2011). TRIM5 is an innate immune sensor for the retrovirus capsid lattice. Nature.

[46] Kutluay SB, Perez-Caballero D, Bieniasz PD (2013). Fates of retroviral core components during unrestricted and TRIM5-restricted infection. PLoS Pathog.

[47] Wu X, Anderson JL, Campbell EM, Joseph AM, Hope TJ (2006). Proteasome inhibitors uncouple rhesus TRIM5alpha restriction of HIV-1 reverse transcription and infection. Proc Natl Acad Sci USA.

[48] Fletcher AJ, Christensen DE, Nelson C, Tan CP, Schaller T (2015). TRIM5α requires Ube2W to anchor Lys63-linked ubiquitin chains and restrict reverse transcription. The EMBO Journal.

[49] Jimenez-Guardeño JM, Apolonia L, Betancor G, Malim MH (2019). Immunoproteasome activation enables human TRIM5α restriction of HIV-1. Nat Microbiol.

[50] Selyutina A, Simons LM, Kirby KA, Bulnes-Ramos A, Hu P (2022). TRIM5α restriction of HIV-1-N74D viruses in lymphocytes is caused by a loss of Cyclophilin A protection. Viruses.

[51] Osega CE, Bustos FJ, Arriagada G (2024). From entry to the nucleus: how retroviruses commute. Annu Rev Virol.

[52] Huang P-T, Summers BJ, Xu C, Perilla JR, Malikov V (2019). FEZ1 is recruited to a conserved cofactor site on capsid to promote HIV-1 trafficking. Cell Reports.

[53] Malikov V, da Silva ES, Jovasevic V, Bennett G, de Souza Aranha Vieira DA (2015). HIV-1 capsids bind and exploit the kinesin-1 adaptor FEZ1 for inward movement to the nucleus. Nat Commun.

[54] Carnes SK, Zhou J, Aiken C (2018). HIV-1 engages a dynein-dynactin-BICD2 complex for infection and transport to the nucleus. J Virol.

[55] Dharan A, Opp S, Abdel-Rahim O, Keceli SK, Imam S (2017). Bicaudal D2 facilitates the cytoplasmic trafficking and nuclear import of HIV-1 genomes during infection. Proc Natl Acad Sci U S A.

[56] Dharan A, Talley S, Tripathi A, Mamede JI, Majetschak M (2016). KIF5B and Nup358 cooperatively mediate the nuclear import of HIV-1 during infection. PLoS Pathog.

[57] Yamashita M, Emerman M (2004). Capsid is a dominant determinant of retrovirus infectivity in nondividing cells. J Virol.

[58] Yamashita M, Perez O, Hope TJ, Emerman M (2007). Evidence for direct involvement of the capsid protein in HIV infection of nondividing cells. PLoS Pathog.

[59] Briggs JAG, Wilk T, Welker R, Kräusslich H-G, Fuller SD (2003). Structural organization of authentic, mature HIV-1 virions and cores. EMBO J.

[60] von Appen A, Kosinski J, Sparks L, Ori A, DiGuilio AL (2015). In situ structural analysis of the human nuclear pore complex. Nature.

[61] Zila V, Margiotta E, Turoňová B, Müller TG, Zimmerli CE (2021). Cone-shaped HIV-1 capsids are transported through intact nuclear pores. Cell.

[62] Bejarano DA, Peng K, Laketa V, Börner K, Jost KL (2019). HIV-1 nuclear import in macrophages is regulated by CPSF6-capsid interactions at the nuclear pore complex. eLife.

[63] Selyutina A, Persaud M, Lee K, KewalRamani V, Diaz-Griffero F (2020). Nuclear import of the HIV-1 core precedes reverse transcription and uncoating. Cell Reports.

[64] Schifferdecker S, Zila V, Müller TG, Sakin V, Anders-Össwein M (2022). Direct capsid labeling of infectious HIV-1 by genetic code expansion allows detection of largely complete nuclear capsids and suggests nuclear entry of HIV-1 complexes via common routes. mBio.

[65] Dharan A, Bachmann N, Talley S, Zwikelmaier V, Campbell EM (2020). Nuclear pore blockade reveals that HIV-1 completes reverse transcription and uncoating in the nucleus. Nat Microbiol.

[66] Peng K, Muranyi W, Glass B, Laketa V, Yant SR (2014). Quantitative microscopy of functional HIV post-entry complexes reveals association of replication with the viral capsid. Elife.

[67] Zila V, Müller TG, Laketa V, Müller B, Kräusslich H-G (2019). Analysis of CA content and CPSF6 dependence of early HIV-1 replication complexes in SupT1-R5 cells. mBio.

[68] Burdick RC, Li C, Munshi M, Rawson JMO, Nagashima K (2020). HIV-1 uncoats in the nucleus near sites of integration. Proc Natl Acad Sci U S A.

[69] Li C, Burdick RC, Nagashima K, Hu W-S, Pathak VK (2021). HIV-1 cores retain their integrity until minutes before uncoating in the nucleus. Proc Natl Acad Sci USA.

[70] Müller TG, Zila V, Peters K, Schifferdecker S, Stanic M (2021). HIV-1 uncoating by release of viral cDNA from capsid-like structures in the nucleus of infected cells. eLife.

[71] Akey CW, Singh D, Ouch C, Echeverria I, Nudelman I (2022). Comprehensive structure and functional adaptations of the yeast nuclear pore complex. Cell.

[72] Mosalaganti S, Obarska-Kosinska A, Siggel M, Taniguchi R, Turoňová B (2022). AI-based structure prediction empowers integrative structural analysis of human nuclear pores. Science.

[73] Petrovic S, Samanta D, Perriches T, Bley CJ, Thierbach K (2022). Architecture of the linker-scaffold in the nuclear pore. Science.

[74] Kim SJ, Fernandez-Martinez J, Nudelman I, Shi Y, Zhang W (2018). Integrative structure and functional anatomy of a nuclear pore complex. Nature.

[75] Hampoelz B, Andres-Pons A, Kastritis P, Beck M (2019). Structure and assembly of the nuclear pore complex. Annu Rev Biophys.

[76] Wing CE, Fung HYJ, Chook YM (2022). Karyopherin-mediated nucleocytoplasmic transport. Nat Rev Mol Cell Biol.

[77] Brass AL, Dykxhoorn DM, Benita Y, Yan N, Engelman A (2008). Identification of host proteins required for HIV infection through a functional genomic screen. Science.

[78] Christ F, Thys W, De Rijck J, Gijsbers R, Albanese A (2008). Transportin-SR2 imports HIV into the nucleus. Curr Biol.

[79] König R, Zhou Y, Elleder D, Diamond TL, Bonamy GMC (2008). Global analysis of host-pathogen interactions that regulate early-stage HIV-1 replication. Cell.

[80] Lee K, Ambrose Z, Martin TD, Oztop I, Mulky A (2010). Flexible use of nuclear import pathways by HIV-1. Cell Host & Microbe.

[81] Maertens GN, Cook NJ, Wang W, Hare S, Gupta SS (2014). Structural basis for nuclear import of splicing factors by human Transportin 3. Proc Natl Acad Sci U S A.

[82] Dickson CF, Hertel S, Tuckwell AJ, Li N, Ruan J (2024). The HIV capsid mimics karyopherin engagement of FG-nucleoporins. Nature.

[83] Fu L, Weiskopf EN, Akkermans O, Swanson NA, Cheng S (2024). HIV-1 capsids enter the FG phase of nuclear pores like a transport receptor. Nature.

[84] Matreyek KA, Yücel SS, Li X, Engelman A (2013). Nucleoporin NUP153 phenylalanine-glycine motifs engage a common binding pocket within the HIV-1 capsid protein to mediate lentiviral infectivity. PLoS Pathog.

[85] Buffone C, Martinez-Lopez A, Fricke T, Opp S, Severgnini M (2018). Nup153 unlocks the nuclear pore complex for HIV-1 nuclear translocation in nondividing cells. J Virol.

[86] Kane M, Rebensburg SV, Takata MA, Zang TM, Yamashita M (2018). Nuclear pore heterogeneity influences HIV-1 infection and the antiviral activity of MX2. Elife.

[87] Müller TG, Zila V, Müller B, Kräusslich H-G (2022). Nuclear capsid uncoating and reverse transcription of HIV-1. Annu Rev Virol.

[88] Achuthan V, Perreira JM, Sowd GA, Puray-Chavez M, McDougall WM (2018). Capsid-CPSF6 interaction licenses nuclear HIV-1 trafficking to sites of viral DNA integration. Cell Host Microbe.

[89] Chin CR, Perreira JM, Savidis G, Portmann JM, Aker AM (2015). Direct visualization of HIV-1 replication intermediates shows that capsid and CPSF6 modulate HIV-1 intra-nuclear invasion and integration. Cell Rep.

[90] Márquez CL, Lau D, Walsh J, Shah V, McGuinness C (2018). Kinetics of HIV-1 capsid uncoating revealed by single-molecule analysis. Elife.

[91] Faysal KR, Walsh JC, Renner N, Márquez CL, Shah VB Pharmacologic hyperstabilisation of the HIV-1 capsid lattice induces capsid failure. eLife.

[92] Christensen DE, Ganser-Pornillos BK, Johnson JS, Pornillos O, Sundquist WI (2020). Reconstitution and visualization of HIV-1 capsid-dependent replication and integration in vitro. *Science*.

[93] Price AJ, Jacques DA, McEwan WA, Fletcher AJ, Essig S (2014). Host cofactors and pharmacologic ligands share an essential interface in HIV-1 capsid that is lost upon disassembly. PLoS Pathog.

[94] Ning J, Zhong Z, Fischer DK, Harris G, Watkins SC (2018). Truncated CPSF6 forms higher-order complexes that bind and disrupt HIV-1 capsid. J Virol.

[95] Zhong Z, Ning J, Boggs EA, Jang S, Wallace C (2021). Cytoplasmic CPSF6 Regulates HIV-1 Capsid Trafficking and Infection in A Cyclophilin A-Dependent Manner. mBio.

[96] Ingram Z, Kline C, Hughson AK, Singh PK, Fischer HL (2024). Spatiotemporal binding of cyclophilin A and CPSF6 to capsid regulates HIV-1 nuclear entry and integration. bioRxiv.

[97] Bhattacharya A, Alam SL, Fricke T, Zadrozny K, Sedzicki J (2014). Structural basis of HIV-1 capsid recognition by PF74 and CPSF6. Proc Natl Acad Sci USA.

[98] Price AJ, Fletcher AJ, Schaller T, Elliott T, Lee K (2012). CPSF6 defines a conserved capsid interface that modulates HIV-1 replication. PLoS Pathog.

[99] Luchsinger C, Lee K, Mardones GA, KewalRamani VN, Diaz-Griffero F (2023). Formation of nuclear CPSF6/CPSF5 biomolecular condensates upon HIV-1 entry into the nucleus is important for productive infection. Sci Rep.

[100] Francis AC, Marin M, Singh PK, Achuthan V, Prellberg MJ (2020). HIV-1 replication complexes accumulate in nuclear speckles and integrate into speckle-associated genomic domains. Nat Commun.

[101] Greig JA, Nguyen TA, Lee M, Holehouse AS, Posey AE (2020). Arginine-enriched mixed-charge domains provide cohesion for nuclear speckle condensation. Mol Cell.

[102] Burdick RC, Morse M, Rouzina I, Williams MC, Hu W-S (2024). HIV-1 uncoating requires long double-stranded reverse transcription products. Sci Adv.

[103] Gifford LB, Melikyan GB (2024). HIV-1 capsid uncoating is a multistep process that proceeds through defect formation followed by disassembly of the capsid lattice. ACS Nano.

[104] Rankovic S, Varadarajan J, Ramalho R, Aiken C, Rousso I (2017). Reverse transcription mechanically initiates HIV-1 capsid disassembly. J Virol.

[105] Hulme AE, Perez O, Hope TJ (2011). Complementary assays reveal a relationship between HIV-1 uncoating and reverse transcription. Proc Natl Acad Sci U S A.

[106] Rankovic S, Deshpande A, Harel S, Aiken C, Rousso I (2021). HIV-1 uncoating occurs via a series of rapid biomechanical changes in the core related to individual stages of reverse transcription. J Virol.

[107] Rensen E, Mueller F, Scoca V, Parmar JJ, Souque P (2021). Clustering and reverse transcription of HIV-1 genomes in nuclear niches of macrophages. EMBO J.

[108] Yang Y, Fricke T, Diaz-Griffero F (2013). Inhibition of reverse transcriptase activity increases stability of the HIV-1 core. J Virol.

[109] Rouzina I, Bruinsma R (2014). DNA confinement drives uncoating of the HIV Virus. Eur Phys J Spec Top.

[110] Schirra RT, Dos Santos NFB, Zadrozny KK, Kucharska I, Ganser-Pornillos BK (2023). A molecular switch modulates assembly and host factor binding of the HIV-1 capsid. Nat Struct Mol Biol.

[111] Renner N, Kleinpeter A, Mallery DL, Albecka A, Rifat Faysal KM (2023). HIV-1 is dependent on its immature lattice to recruit IP6 for mature capsid assembly. Nat Struct Mol Biol.

[112] Mallery DL, Faysal KMR, Kleinpeter A, Wilson MSC, Vaysburd M (2019). Cellular IP6 levels limit HIV-1 production while viruses that cannot efficiently package IP6 are attenuated for infection and replication. Cell Rep.

[113] Mallery DL, Kleinpeter AB, Renner N, Faysal KMR, Novikova M (2021). A stable immature lattice packages IP6 for HIV capsid maturation. Sci Adv.

[114] Lu M, Hou G, Zhang H, Suiter CL, Ahn J (2015). Dynamic allostery governs cyclophilin A-HIV capsid interplay. Proc Natl Acad Sci U S A.

[115] Perilla JR, Hadden JA, Goh BC, Mayne CG, Schulten K (2016). All-atom molecular dynamics of virus capsids as drug targets. J Phys Chem Lett.

[116] Blair WS, Pickford C, Irving SL, Brown DG, Anderson M (2010). HIV-1 capsid is a tractable target for small molecule therapeutic intervention. PLoS Pathog.

[117] Shi J, Zhou J, Shah VB, Aiken C, Whitby K (2011). Small-molecule inhibition of human immunodeficiency virus type 1 infection by virus capsid destabilization. J Virol.

[118] Wang C, Huang H, Mallon K, Valera L, Parcella K (2023). Antiviral properties of HIV-1 capsid inhibitor GSK878. Antimicrob Agents Chemother.

[119] Parra RG, Schafer NP, Radusky LG, Tsai M-Y, Guzovsky AB (2016). Protein Frustratometer 2: a tool to localize energetic frustration in protein molecules, now with electrostatic. Nucleic Acids Res.

[120] Ferreiro DU, Komives EA, Wolynes PG (2018). Frustration, function and folding. Curr Opin Struct Biol.

[121] Chen M, Chen X, Schafer NP, Clementi C, Komives EA (2020). Surveying biomolecular frustration at atomic resolution. Nat Commun.

[122] Twarock R, Towers GJ, Stockley PG (2024). Molecular frustration: a hypothesis for regulation of viral infections. Trends Microbiol.

[123] Ni T, Zhu Y, Yang Z, Xu C, Chaban Y (2021). Structure of native HIV-1 cores and their interactions with IP6 and CypA. Sci Adv.

[124] Sauter D, Kirchhoff F (2019). Key viral adaptations preceding the AIDS pandemic. Cell Host Microbe.

[125] World Health Organisation HIV. https://www.who.int/data/gho/data/themes/hiv-aids#:~:text=Since%20the%20beginning%20of%20the,at%20the%20end%20of%202022.

[126] Marlink R, Kanki P, Thior I, Travers K, Eisen G (1994). Reduced rate of disease development after HIV-2 infection as compared to HIV-1. Science.

[127] O’Donovan D, Ariyoshi K, Milligan P, Ota M, Yamuah L (2000). Maternal plasma viral RNA levels determine marked differences in mother-to-child transmission rates of HIV-1 and HIV-2 in The Gambia. MRC/Gambia Government/University College London Medical School working group on mother-child transmission of HIV. AIDS.

[128] Andersson S, Norrgren H, da Silva Z, Biague A, Bamba S (2000). Plasma viral load in HIV-1 and HIV-2 singly and dually infected individuals in Guinea-Bissau, West Africa: significantly lower plasma virus set point in HIV-2 infection than in HIV-1 infection. Arch Intern Med.

[129] Sandler NG, Bosinger SE, Estes JD, Zhu RTR, Tharp GK (2014). Type I interferon responses in rhesus macaques prevent SIV infection and slow disease progression. Nature.

[130] Lahaye X, Satoh T, Gentili M, Cerboni S, Conrad C (2013). The capsids of HIV-1 and HIV-2 determine immune detection of the viral cDNA by the innate sensor cGAS in dendritic cells. Immunity.

[131] Manel N, Hogstad B, Wang Y, Levy DE, Unutmaz D (2010). A cryptic sensor for HIV-1 activates antiviral innate immunity in dendritic cells. Nature.

[132] Zuliani-Alvarez L, Govasli ML, Rasaiyaah J, Monit C, Perry SO (2022). Evasion of cGAS and TRIM5 defines pandemic HIV. Nat Microbiol.

[133] Marchant D, Neil SJD, McKnight Á (2006). Human immunodeficiency virus types 1 and 2 have different replication kinetics in human primary macrophage culture. J Gen Virol.

[134] Santos-Costa Q, Lopes MM, Calado M, Azevedo-Pereira JM (2014). HIV-2 interaction with cell coreceptors: amino acids within the V1/V2 region of viral envelope are determinant for CCR8, CCR5 and CXCR4 usage. Retrovirology.

[135] Bron R, Klasse PJ, Wilkinson D, Clapham PR, Pelchen-Matthews A (1997). Promiscuous use of CC and CXC chemokine receptors in cell-to-cell fusion mediated by a human immunodeficiency virus type 2 envelope protein. J Virol.

[136] Reeves JD, Hibbitts S, Simmons G, McKnight A, Azevedo-Pereira JM (1999). Primary human immunodeficiency virus type 2 (HIV-2) isolates infect CD4-negative cells via CCR5 and CXCR4: comparison with HIV-1 and simian immunodeficiency virus and relevance to cell tropism in vivo. J Virol.

[137] McKnight A, Clapham PR, Weiss RA (1994). HIV-2 and SIV infection of nonprimate cell lines expressing human CD4: restrictions to replication at distinct stages. Virology.

[138] Yamashita M, Emerman M (2006). Retroviral infection of non-dividing cells: old and new perspectives. Virology.

[139] Mamede JI, Damond F, Bernardo A de, Matheron S, Descamps D (2017). Cyclophilins and nucleoporins are required for infection mediated by capsids from circulating HIV-2 primary isolates. Sci Rep.

[140] Li W, Singh PK, Sowd GA, Bedwell GJ, Jang S (2020). CPSF6-dependent targeting of speckle-associated domains distinguishes primate from nonprimate lentiviral integration in HIV-1. mBio.

[141] Rebensburg SV, Wei G, Larue RC, Lindenberger J, Francis AC (2021). Sec24C is an HIV-1 host dependency factor crucial for virus replication. Nat Microbiol.

[142] Ylinen LMJ, Price AJ, Rasaiyaah J, Hué S, Rose NJ (2010). Conformational adaptation of Asian macaque TRIMCyp directs lineage specific antiviral activity. PLoS Pathog.

[143] Price AJ, Marzetta F, Lammers M, Ylinen LMJ, Schaller T (2009). Active site remodeling switches HIV specificity of antiretroviral TRIMCyp. Nat Struct Mol Biol.

[144] Jacques DA, McEwan WA, Hilditch L, Price AJ, Towers GJ (2016). HIV-1 uses dynamic capsid pores to import nucleotides and fuel encapsidated DNA synthesis. Nature.

[145] Twizerimana AP, Becker D, Zhu S, Luedde T, Gohlke H (2023). The cyclophilin A-binding loop of the capsid regulates the human TRIM5α sensitivity of nonpandemic HIV-1. Proc Natl Acad Sci U S A.

[146] Morellet N, Bouaziz S, Petitjean P, Roques BP (2003). NMR structure of the HIV-1 regulatory protein VPR. J Mol Biol.

[147] Khamsri B, Murao F, Yoshida A, Sakurai A, Uchiyama T (2006). Comparative study on the structure and cytopathogenic activity of HIV Vpr/Vpx proteins. Microbes and Infection.

[148] Miyatake H, Sanjoh A, Murakami T, Murakami H, Matsuda G (2016). Molecular mechanism of HIV-1 Vpr for binding to importin-α. J Mol Biol.

[149] Selig L, Pages J-C, Tanchou V, Prévéral S, Berlioz-Torrent C (1999). Interaction with the p6 domain of the Gag precursor mediates incorporation into virions of Vpr and Vpx proteins from primate lentiviruses. J Virol.

[150] Desai TM, Marin M, Sood C, Shi J, Nawaz F (2015). Fluorescent protein-tagged Vpr dissociates from HIV-1 core after viral fusion and rapidly enters the cell nucleus. Retrovirology.

[151] Belshan M, Ratner L (2003). Identification of the nuclear localization signal of human immunodeficiency virus type 2 Vpx. Virology.

[152] Tristem M, Purvis A, Quicke DLJ (1998). Complex evolutionary history of primate lentiviral vpr genes. Virology.

[153] McCarthy KR, Johnson WE (2014). Plastic proteins and monkey blocks: how lentiviruses evolved to replicate in the presence of primate restriction factors. PLoS Pathog.

[154] Romani B, Cohen EA (2012). Lentivirus Vpr and Vpx accessory proteins usurp the cullin4-DDB1 (DCAF1) E3 ubiquitin ligase. Curr Opin Virol.

[155] Greenwood EJD, Williamson JC, Sienkiewicz A, Naamati A, Matheson NJ (2019). Promiscuous targeting of cellular proteins by Vpr drives systems-level proteomic remodeling in HIV-1 infection. Cell Reports.

[156] Schwefel D, Groom HCT, Boucherit VC, Christodoulou E, Walker PA (2014). Structural basis of lentiviral subversion of a cellular protein degradation pathway. Nature.

[157] Schwefel D, Boucherit VC, Christodoulou E, Walker PA, Stoye JP (2015). Molecular determinants for recognition of divergent SAMHD1 proteins by the lentiviral accessory protein Vpx. Cell Host Microbe.

[158] Laguette N, Sobhian B, Casartelli N, Ringeard M, Chable-Bessia C (2011). SAMHD1 is the dendritic- and myeloid-cell-specific HIV-1 restriction factor counteracted by Vpx. Nature.

[159] Hofmann H, Logue EC, Bloch N, Daddacha W, Polsky SB (2012). The Vpx lentiviral accessory protein targets SAMHD1 for degradation in the nucleus. J Virol.

[160] Goldstone DC, Ennis-Adeniran V, Hedden JJ, Groom HCT, Rice GI (2011). HIV-1 restriction factor SAMHD1 is a deoxynucleoside triphosphate triphosphohydrolase. Nature.

[161] Lahouassa H, Daddacha W, Hofmann H, Ayinde D, Logue EC (2012). SAMHD1 restricts the replication of human immunodeficiency virus type 1 by depleting the intracellular pool of deoxynucleoside triphosphates. Nat Immunol.

[162] Mlcochova P, Sutherland KA, Watters SA, Bertoli C, de Bruin RA (2017). A G1-like state allows HIV-1 to bypass SAMHD1 restriction in macrophages. EMBO J.

[163] Schott K, Fuchs NV, Derua R, Mahboubi B, Schnellbächer E (2018). Dephosphorylation of the HIV-1 restriction factor SAMHD1 is mediated by PP2A-B55α holoenzymes during mitotic exit. Nat Commun.

[164] Cribier A, Descours B, Valadão ALC, Laguette N, Benkirane M (2013). Phosphorylation of SAMHD1 by Cyclin A2/CDK1 regulates its restriction activity toward HIV-1. Cell Reports.

[165] White TE, Brandariz-Nuñez A, Valle-Casuso JC, Amie S, Nguyen LA (2013). The retroviral restriction ability of SAMHD1, but not its deoxynucleotide triphosphohydrolase activity, is regulated by phosphorylation. Cell Host Microbe.

[166] Hrecka K, Hao C, Gierszewska M, Swanson SK, Kesik-Brodacka M (2011). Vpx relieves inhibition of HIV-1 infection of macrophages mediated by the SAMHD1 protein. Nature.

[167] Sunseri N, O’Brien M, Bhardwaj N, Landau NR (2011). Human immunodeficiency virus type 1 modified to package simian immunodeficiency virus Vpx efficiently infects macrophages and dendritic cells. J Virol.

[168] Yurkovetskiy L, Guney MH, Kim K, Goh SL, McCauley S (2018). Primate immunodeficiency virus proteins Vpx and Vpr counteract transcriptional repression of proviruses by the HUSH complex. Nat Microbiol.

[169] Goujon C, Jarrosson-Wuillème L, Bernaud J, Rigal D, Darlix J-L (2006). With a little help from a friend: increasing HIV transduction of monocyte-derived dendritic cells with virion-like particles of SIV(MAC). Gene Ther.

[170] Duvall MG, Loré K, Blaak H, Ambrozak DA, Adams WC (2007). Dendritic cells are less susceptible to human immunodeficiency virus type 2 (HIV-2) infection than to HIV-1 infection. J Virol.

[171] Chauveau L, Puigdomenech I, Ayinde D, Roesch F, Porrot F (2015). HIV-2 infects resting CD4+ T cells but not monocyte-derived dendritic cells. Retrovirology.

[172] Baldauf H-M, Pan X, Erikson E, Schmidt S, Daddacha W (2012). SAMHD1 restricts HIV-1 infection in resting CD4(+) T cells. Nat Med.

[173] Samri A, Charpentier C, Diallo MS, Bertine M, Even S (2019). Limited HIV-2 reservoirs in central-memory CD4 T-cells associated to CXCR6 co-receptor expression in attenuated HIV-2 infection. PLoS Pathog.

[174] Tchasovnikarova IA, Timms RT, Matheson NJ, Wals K, Antrobus R (2015). GENE SILENCING. epigenetic silencing by the HUSH complex mediates position-effect variegation in human cells. Science.

[175] Seczynska M, Bloor S, Cuesta SM, Lehner PJ (2022). Genome surveillance by HUSH-mediated silencing of intronless mobile elements. Nature.

[176] Timms RT, Tchasovnikarova IA, Antrobus R, Dougan G, Lehner PJ (2016). ATF7IP-mediated stabilization of the histone methyltransferase SETDB1 is essential for heterochromatin formation by the HUSH complex. Cell Rep.

[177] Müller I, Helin K (2024). Keep quiet: the HUSH complex in transcriptional silencing and disease. Nat Struct Mol Biol.

[178] Garland W, Müller I, Wu M, Schmid M, Imamura K (2022). Chromatin modifier HUSH co-operates with RNA decay factor NEXT to restrict transposable element expression. Mol Cell.

[179] Matkovic R, Morel M, Lanciano S, Larrous P, Martin B (2022). TASOR epigenetic repressor cooperates with a CNOT1 RNA degradation pathway to repress HIV. Nat Commun.

[180] Chougui G, Munir-Matloob S, Matkovic R, Martin MM, Morel M (2018). HIV-2/SIV viral protein X counteracts HUSH repressor complex. Nat Microbiol.

[181] Tunbak H, Enriquez-Gasca R, Tie CHC, Gould PA, Mlcochova P (2020). The HUSH complex is a gatekeeper of type I interferon through epigenetic regulation of LINE-1s. Nat Commun.

[182] Danac JMC, Matthews RE, Gungi A, Qin C, Parsons H (2024). Competition between two HUSH complexes orchestrates the immune response to retroelement invasion. Mol Cell.

[183] Laliberté A, Prelli Bozzo C, Stahl-Hennig C, Hunszinger V, Joas S (2023). Vpr attenuates antiviral immune responses and is critical for full pathogenicity of SIV_mac239_ in rhesus macaques. iScience.

[184] Ali A, Ng HL, Blankson JN, Burton DR, Buckheit RW (2018). Highly attenuated infection with a Vpr-deleted molecular clone of Human immunodeficiency virus-1. J Infect Dis.

[185] Balliet JW, Kolson DL, Eiger G, Kim FM, McGann KA (1994). Distinct effects in primary macrophages and lymphocytes of the human immunodeficiency virus type 1 accessory genes vpr, vpu, and nef: mutational analysis of a primary HIV-1 isolate. Virology.

[186] Connor RI, Chen BK, Choe S, Landau NR (1995). Vpr is required for efficient replication of human immunodeficiency virus type-1 in mononuclear phagocytes. Virology.

[187] Harman AN, Nasr N, Feetham A, Galoyan A, Alshehri AA (2015). HIV blocks interferon induction in human dendritic cells and macrophages by dysregulation of TBK1. J Virol.

[188] Lubow J, Collins KL (2020). Vpr is a VIP: HIV Vpr and infected macrophages promote viral pathogenesis. Viruses.

[189] Hattori N, Michaels F, Fargnoli K, Marcon L, Gallo RC (1990). The human immunodeficiency virus type 2 vpr gene is essential for productive infection of human macrophages. Proc Natl Acad Sci U S A.

[190] Vermeire J, Roesch F, Sauter D, Rua R, Hotter D (2016). HIV triggers a cGAS-dependent, Vpu- and Vpr-regulated type I interferon response in CD4^+^ T Cells. Cell Rep.

[191] Khan H, Sumner RP, Rasaiyaah J, Tan CP, Rodriguez-Plata MT HIV-1 Vpr antagonizes innate immune activation by targeting karyopherin-mediated NF-κB/IRF3 nuclear transport. eLife.

[192] Reuschl A-K, Mesner D, Shivkumar M, Whelan MVX, Pallett LJ (2022). HIV-1 Vpr drives a tissue residency-like phenotype during selective infection of resting memory T cells. Cell Rep.

[193] Vodicka MA, Koepp DM, Silver PA, Emerman M (1998). HIV-1 Vpr interacts with the nuclear transport pathway to promote macrophage infection. Genes Dev.

[194] Forouzanfar F, Ali S, Wallet C, De Rovere M, Ducloy C (2019). HIV-1 Vpr mediates the depletion of the cellular repressor CTIP2 to counteract viral gene silencing. Sci Rep.

[195] Kawano K, Doucet AJ, Ueno M, Kariya R, An W (2018). HIV-1 Vpr and p21 restrict LINE-1 mobility. Nucleic Acids Res.

[196] Pace MJ, Graf EH, O’Doherty U (2013). HIV 2-long terminal repeat circular DNA is stable in primary CD4+T Cells. Virology.

[197] Richetta C, Thierry S, Thierry E, Lesbats P, Lapaillerie D (2019). Two-long terminal repeat (LTR) DNA circles are a substrate for HIV-1 integrase. J Biol Chem.

[198] Thierry S, Munir S, Thierry E, Subra F, Leh H (2015). Integrase inhibitor reversal dynamics indicate unintegrated HIV-1 dna initiate de novo integration. Retrovirology.

[199] Munir S, Thierry S, Subra F, Deprez E, Delelis O (2013). Quantitative analysis of the time-course of viral DNA forms during the HIV-1 life cycle. Retrovirology.

[200] Dupont L, Bloor S, Williamson JC, Cuesta SM, Shah R (2021). The SMC5/6 complex compacts and silences unintegrated HIV-1 DNA and is antagonized by Vpr. Cell Host Microbe.

[201] Irwan ID, Bogerd HP, Cullen BR (2022). Epigenetic silencing by the SMC5/6 complex mediates HIV-1 latency. Nat Microbiol.

[202] Roshal M, Kim B, Zhu Y, Nghiem P, Planelles V (2003). Activation of the ATR-mediated DNA damage response by the HIV-1 viral protein R. J Biol Chem.

[203] Planelles V, Jowett JB, Li QX, Xie Y, Hahn B (1996). Vpr-induced cell cycle arrest is conserved among primate lentiviruses. J Virol.

[204] Laguette N, Brégnard C, Hue P, Basbous J, Yatim A (2014). Premature activation of the SLX4 complex by Vpr promotes G2/M arrest and escape from innate immune sensing. Cell.

[205] Li D, Lopez A, Sandoval C, Nichols Doyle R, Fregoso OI (2020). HIV Vpr Modulates the Host DNA Damage Response at Two Independent Steps to Damage DNA and Repress Double-Strand DNA Break Repair. mBio.

[206] Zhang F, Bieniasz PD (2020). HIV-1 vpr induces cell cycle arrest and enhances viral gene expression by depleting CCDC137. Elife.

[207] Fekairi S, Scaglione S, Chahwan C, Taylor ER, Tissier A (2009). Human SLX4 is a holliday junction resolvase subunit that binds multiple DNA repair/recombination endonucleases. Cell.

[208] Belzile J-P, Abrahamyan LG, Gérard FCA, Rougeau N, Cohen EA (2010). Formation of mobile chromatin-associated nuclear foci containing HIV-1 Vpr and VPRBP is critical for the induction of G2 cell cycle arrest. PLoS Pathog.

[209] Hrecka K, Hao C, Shun M-C, Kaur S, Swanson SK (2016). HIV-1 and HIV-2 exhibit divergent interactions with HLTF and UNG2 DNA repair proteins. Proc Natl Acad Sci U S A.

[210] Li D, Wu M (2021). Pattern recognition receptors in health and diseases. Signal Transduct Target Ther.

[211] Ma Z, Damania B (2016). The cGAS-STING defense pathway and its counteraction by viruses. Cell Host Microbe.

[212] Müller U, Steinhoff U, Reis LF, Hemmi S, Pavlovic J (1994). Functional role of type I and type II interferons in antiviral defense. Science.

[213] Iwasaki A, Medzhitov R (2015). Control of adaptive immunity by the innate immune system. Nat Immunol.

[214] Schneider WM, Chevillotte MD, Rice CM (2014). Interferon-stimulated genes: a complex web of host defenses. Annu Rev Immunol.

[215] Schmidt N, Domingues P, Golebiowski F, Patzina C, Tatham MH (2019). An influenza virus-triggered SUMO switch orchestrates co-opted endogenous retroviruses to stimulate host antiviral immunity. Proc Natl Acad Sci U S A.

[216] Rookhuizen DC, Bonte P-E, Ye M, Hoyler T, Gentili M Induction of transposable element expression is central to innate sensing. *Immunology*.

[217] Li H-T, Jang HJ, Rohena-Rivera K, Liu M, Gujar H (2023). RNA mis-splicing drives viral mimicry response after DNMTi therapy in SETD2-mutant kidney cancer. Cell Rep.

[218] Briones MS, Dobard CW, Chow SA (2010). Role of human immunodeficiency virus type 1 integrase in uncoating of the viral core. J Virol.

[219] Liu Z, Pan Q, Ding S, Qian J, Xu F (2013). The interferon-inducible MxB protein inhibits HIV-1 infection. Cell Host Microbe.

[220] Franke EK, Yuan HE, Luban J (1994). Specific incorporation of cyclophilin A into HIV-1 virions. Nature.

[221] Thali M, Bukovsky A, Kondo E, Rosenwirth B, Walsh CT (1994). Functional association of cyclophilin A with HIV-1 virions. Nature.

[222] Vajdos FF, Yoo S, Houseweart M, Sundquist WI, Hill CP (1997). Crystal structure of cyclophilin A complexed with A binding site peptide from the HIV-1 capsid protein. Protein Sci.

[223] Luban J, Bossolt KL, Franke EK, Kalpana GV, Goff SP (1993). Human immunodeficiency virus type 1 Gag protein binds to cyclophilins A and B. Cell.

[224] Bosco DA, Eisenmesser EZ, Pochapsky S, Sundquist WI, Kern D (2002). Catalysis of cis/trans isomerization in native HIV-1 capsid by human cyclophilin A. Proc Natl Acad Sci U S A.

[225] Liu C, Perilla JR, Ning J, Lu M, Hou G (2016). Cyclophilin A stabilizes the HIV-1 capsid through A novel non-canonical binding site. Nat Commun.

[226] Shah VB, Shi J, Hout DR, Oztop I, Krishnan L (2013). The host proteins transportin SR2/TNPO3 and cyclophilin A exert opposing effects on HIV-1 uncoating. J Virol.

[227] Keckesova Z, Ylinen LMJ, Towers GJ (2006). Cyclophilin A renders human immunodeficiency virus type 1 sensitive to Old World monkey but not human TRIM5 alpha antiviral activity. J Virol.

[228] Bichel K, Price AJ, Schaller T, Towers GJ, Freund SMV (2013). HIV-1 capsid undergoes coupled binding and isomerization by the nuclear pore protein NUP358. Retrovirology.

[229] Lin DH, Zimmermann S, Stuwe T, Stuwe E, Hoelz A (2013). Structural and functional analysis of the C-terminal domain of Nup358/RanBP2. J Mol Biol.

[230] Matreyek KA, Engelman A (2011). The requirement for nucleoporin NUP153 during human immunodeficiency virus type 1 infection is determined by the viral capsid. J Virol.

[231] Wei G, Iqbal N, Courouble VV, Francis AC, Singh PK (2022). Prion-like low complexity regions enable avid virus-host interactions during HIV-1 infection. Nat Commun.

[232] Stacey JCV, Tan A, Lu JM, James LC, Dick RA (2023). Two structural switches in HIV-1 capsid regulate capsid curvature and host factor binding. Proc Natl Acad Sci U S A.

[233] Scoca V, Morin R, Collard M, Tinevez J-Y, Di Nunzio F (2023). HIV-induced membraneless organelles orchestrate post-nuclear entry steps. J Mol Cell Biol.

[234] Guedán A, Donaldson CD, Caroe ER, Cosnefroy O, Taylor IA (2021). HIV-1 requires capsid remodelling at the nuclear pore for nuclear entry and integration. PLoS Pathog.

[235] Di Nunzio F, Uversky VN, Mouland AJ (2023). Biomolecular condensates: insights into early and late steps of the HIV-1 replication cycle. Retrovirology.

[236] Jang S, Bedwell GJ, Singh SP, Yu HJ, Arnarson B (2024). HIV-1 usurps mixed-charge domain-dependent CPSF6 phase separation for higher-order capsid binding, nuclear entry and viral DNA integration. Nucleic Acids Res.

[237] Link JO, Rhee MS, Tse WC, Zheng J, Somoza JR (2020). Clinical targeting of HIV capsid protein with a long-acting small molecule. Nature.

[238] Bester SM, Wei G, Zhao H, Adu-Ampratwum D, Iqbal N (2020). Structural and mechanistic bases for a potent HIV-1 capsid inhibitor. Science.

[239] Pornillos O, Ganser-Pornillos BK, Yeager M (2011). Atomic-level modelling of the HIV capsid. Nature.

[240] Dick RA, Zadrozny KK, Xu C, Schur FKM, Lyddon TD (2018). Inositol phosphates are assembly co-factors for HIV-1. Nature.

[241] Mallery DL, Márquez CL, McEwan WA, Dickson CF, Jacques DA (2018). IP6 is an HIV pocket factor that prevents capsid collapse and promotes DNA synthesis. Elife.

[242] Xu C, Fischer DK, Rankovic S, Li W, Dick RA (2020). Permeability of the HIV-1 capsid to metabolites modulates viral DNA synthesis. PLoS Biol.

[243] Renner N, Mallery DL, Faysal KMR, Peng W, Jacques DA (2021). A lysine ring in HIV capsid pores coordinates IP6 to drive mature capsid assembly. PLoS Pathog.

[244] Highland CM, Tan A, Ricaña CL, Briggs JAG, Dick RA (2023). Structural insights into HIV-1 polyanion-dependent capsid lattice formation revealed by single particle cryo-EM. Proc Natl Acad Sci U S A.

[245] Gres AT, Kirby KA, McFadden WM, Du H, Liu D (2023). Multidisciplinary studies with mutated HIV-1 capsid proteins reveal structural mechanisms of lattice stabilization. Nat Commun.

[246] Piacentini J, Allen DS, Ganser-Pornillos BK, Chanda SK, Yoh SM (2024). Molecular determinants of PQBP1 binding to the HIV-1 capsid lattice. J Mol Biol.

[247] Yoh SM, Mamede JI, Lau D, Ahn N, Sánchez-Aparicio MT (2022). Recognition of HIV-1 capsid by PQBP1 licenses an innate immune sensing of nascent HIV-1 DNA. Mol Cell.

[248] Yoh SM, Schneider M, Seifried J, Soonthornvacharin S, Akleh RE (2015). PQBP1 is a proximal sensor of the cGAS-dependent innate response to HIV-1. Cell.

[249] Shen Q, Kumari S, Xu C, Jang S, Shi J (2023). The capsid lattice engages a bipartite NUP153 motif to mediate nuclear entry of HIV-1 cores. Proc Natl Acad Sci USA.

[250] Smaga SS, Xu C, Summers BJ, Digianantonio KM, Perilla JR (2019). MxB restricts HIV-1 by targeting the tri-hexamer interface of the viral capsid. Structure.

[251] Goujon C, Greenbury RA, Papaioannou S, Doyle T, Malim MH (2015). A triple-arginine motif in the amino-terminal domain and oligomerization are required for HIV-1 inhibition by human MX2. J Virol.

[252] Busnadiego I, Kane M, Rihn SJ, Preugschas HF, Hughes J (2014). Host and viral determinants of Mx2 antiretroviral activity. J Virol.

[253] Goujon C, Moncorgé O, Bauby H, Doyle T, Ward CC (2013). Human MX2 is an interferon-induced post-entry inhibitor of HIV-1 infection. Nature.

[254] Kane M, Yadav SS, Bitzegeio J, Kutluay SB, Zang T (2013). MX2 is an interferon-induced inhibitor of HIV-1 infection. Nature.

[255] Pornillos O, Ganser-Pornillos BK, Kelly BN, Hua Y, Whitby FG (2009). X-ray structures of the hexameric building block of the HIV capsid. Cell.

[256] Johnson GT, Goodsell DS, Autin L, Forli S, Sanner MF (2014). 3D molecular models of whole HIV-1 virions generated with cellPACK. Faraday Discuss.

[257] Neil S, Martin F, Ikeda Y, Collins M (2001). Postentry restriction to human immunodeficiency virus-based vector transduction in human monocytes. J Virol.

[258] Mlcochova P, Winstone H, Zuliani-Alvarez L, Gupta RK (2020). TLR4-mediated pathway triggers interferon-independent G0 arrest and antiviral SAMHD1 activity in macrophages. Cell Rep.

